# Integrative epigenomics, transcriptomics and proteomics of patient chondrocytes reveal genes and pathways involved in osteoarthritis

**DOI:** 10.1038/s41598-017-09335-6

**Published:** 2017-08-21

**Authors:** Julia Steinberg, Graham R. S. Ritchie, Theodoros I. Roumeliotis, Raveen L. Jayasuriya, Matthew J. Clark, Roger A. Brooks, Abbie L. A. Binch, Karan M. Shah, Rachael Coyle, Mercedes Pardo, Christine L. Le Maitre, Yolande F. M. Ramos, Rob G. H. H. Nelissen, Ingrid Meulenbelt, Andrew W. McCaskie, Jyoti S. Choudhary, J. Mark Wilkinson, Eleftheria Zeggini

**Affiliations:** 1 Wellcome Trust Sanger Institute, Wellcome Trust Genome Campus, Hinxton, Cambridge, CB10 1SA UK; 2 European Molecular Biology Laboratory, European Bioinformatics Institute, Wellcome Trust Genome Campus, Hinxton, Cambridge, CB10 1SD UK; 3 Usher Institute of Population Health Sciences & Informatics, University of Edinburgh, Edinburgh, EH16 4UX UK; 4 MRC Institute of Genetics & Molecular Medicine, University of Edinburgh, Edinburgh, EH4 2XU UK; 5 Department of Oncology and Metabolism, University of Sheffield, Beech Hill Road, Sheffield, S10 2RX UK; 6 Division of Trauma & Orthopaedic Surgery, University of Cambridge, Box 180, Addenbrooke’s Hospital, Hills Road, Cambridge, CB2 0QQ UK; 7 Biomolecular Sciences Research Centre, Sheffield Hallam University, Sheffield, S1 1WB UK; 8 Department of Medical Statistics and Bioinformatics, Section Molecular Epidemiology, Leiden University Medical Center, Leiden, 2300RC The Netherlands; 9 Department of Orthopedics, Leiden University Medical Center, Leiden, 2300RC The Netherlands; 10 Cancer Research Division, Present Address: Cancer Council NSW, Sydney, NSW, 2011 Australia

## Abstract

Osteoarthritis (OA) is a common disease characterized by cartilage degeneration and joint remodeling. The underlying molecular changes underpinning disease progression are incompletely understood. We investigated genes and pathways that mark OA progression in isolated primary chondrocytes taken from paired intact versus degraded articular cartilage samples across 38 patients undergoing joint replacement surgery (discovery cohort: 12 knee OA, replication cohorts: 17 knee OA, 9 hip OA patients). We combined genome-wide DNA methylation, RNA sequencing, and quantitative proteomics data. We identified 49 genes differentially regulated between intact and degraded cartilage in at least two –omics levels, 16 of which have not previously been implicated in OA progression. Integrated pathway analysis implicated the involvement of extracellular matrix degradation, collagen catabolism and angiogenesis in disease progression. Using independent replication datasets, we showed that the direction of change is consistent for over 90% of differentially expressed genes and differentially methylated CpG probes. *AQP1*, *COL1A1* and *CLEC3B* were significantly differentially regulated across all three –omics levels, confirming their differential expression in human disease. Through integration of genome-wide methylation, gene and protein expression data in human primary chondrocytes, we identified consistent molecular players in OA progression that replicated across independent datasets and that have translational potential.

## Introduction

Osteoarthritis (OA) affects in excess of 40% of individuals over the age of 70 years^1^, and is a leading cause of pain and loss of physical function^2^. The molecular mechanisms underlying OA remain incompletely understood and there is no curative therapy for OA; disease progression culminates in joint replacement surgery. OA is a complex disease, with both heritable and environmental factors contributing to susceptibility^3^.

**Figure 1:**
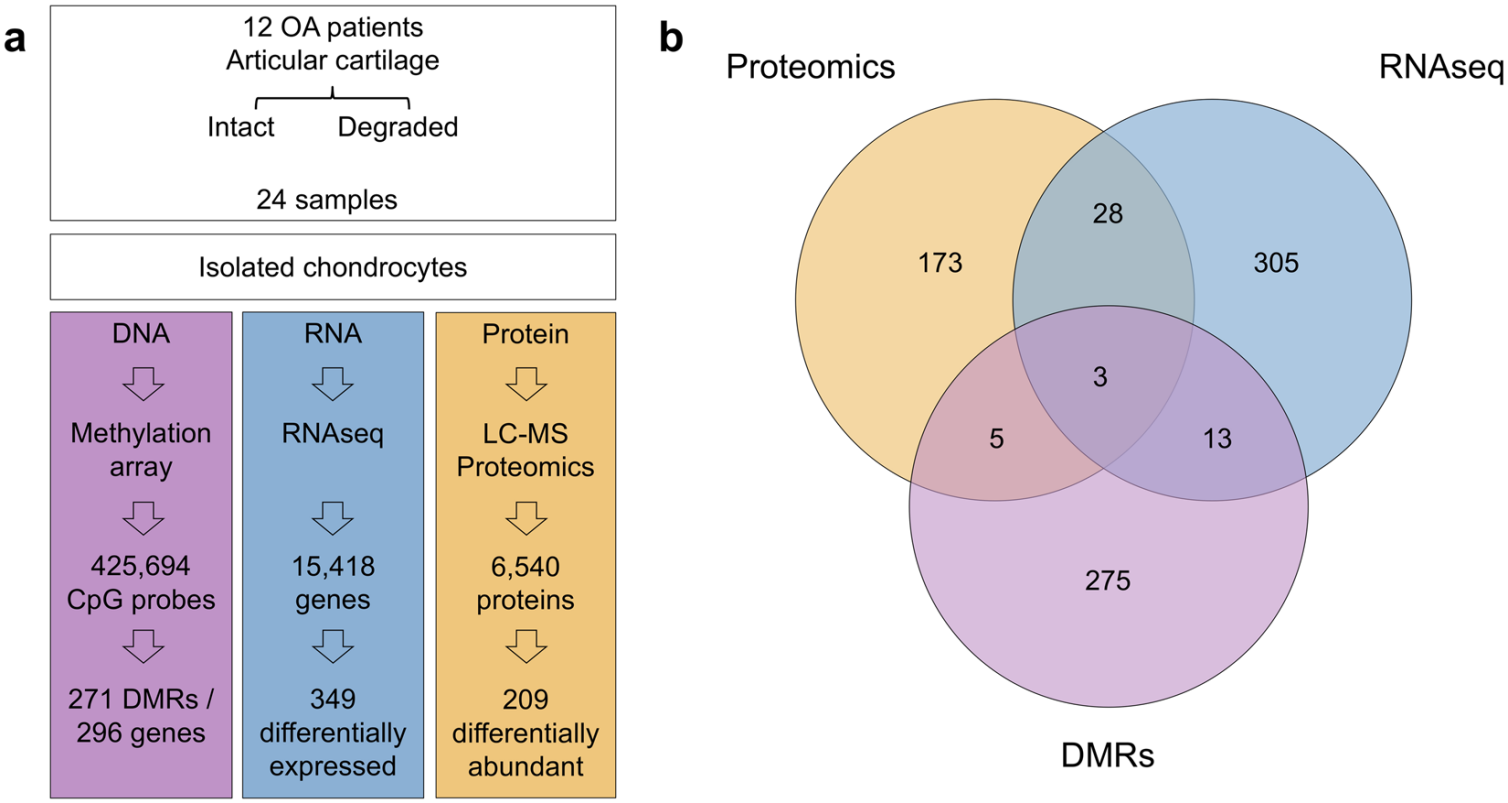
Overview of the genes identified in each –omics experiment and their overlap. (**a**) Schematic view of the 3 functional genomics experiments identifying the number of genes shortlisted for each. (**b**) Venn diagram identifying the number of overlapping shortlisted genes from each individual experiment.

Cartilage degeneration is one of the key features of OA. Cartilage tissue is readily accessible at joint replacement surgery, providing an opportunity to characterize the molecular processes underpinning disease development in the right tissue, both to improve our fundamental understanding of disease biology and to identify novel therapeutic opportunities. In recent years, –omics studies in OA have expanded our understanding of disease pathogenesis, reviewed in refs 4–7. Here we apply integrated multi-omics (DNA CpG methylation, RNA sequencing and quantitative proteomics) in knee OA tissue to obtain a comprehensive molecular portrait of cartilage degeneration (Fig. 1a). The fundamental question here addresses the biological processes underpinning disease progression within the OA joint, which is of direct clinical relevance to patients suffering from OA. To achieve this, we collected individually-matched pairs of cartilage tissue from patients undergoing joint replacement surgery, with one sample demonstrating advanced degenerative change and the other demonstrating little or no evidence of cartilage degeneration. The findings were then replicated in several independent populations of patients undergoing joint replacement. By integrating all three of methylation, gene expression, and protein abundance data, we discover disease processes with involvement across multiple levels, and reveal novel and robustly replicating molecular players with translational potential.

## Methods

For full details of methods see Supplementary Methods.

### Patients and samples

Osteochondral samples were collected from 38 patients undergoing total joint replacement for OA (discovery cohort: 12 knee OA patients; 2 separate replication cohorts: 17 knee OA patients, 9 hip OA patients). Cartilage was separated from bone and chondrocytes were extracted from each sample. DNA, RNA, and protein were extracted from the isolated chondrocytes of each sample. All patients provided full written informed consent prior to participation. We obtained two cartilage samples from each patient. Knee samples were scored using the OARSI cartilage classification system^8, 9^: one sample with high OARSI grade signifying high-grade degeneration (“degraded sample”), and one sample with low OARSI grade signifying healthy tissue or low-grade degeneration (“intact sample”) (Supplementary Fig. S1). Hip samples were classified macroscopically and visually as high-grade (“degraded”) or low-grade (“intact”), details see Supplementary Methods.

### Proteomics (discovery cohort)

LC-MS analysis was performed on the Dionex Ultimate 3000 UHPLC system coupled with the high-resolution LTQ Orbitrap Velos mass spectrometer. Proteins were defined as differentially abundant if the absolute median abundance ratio between degraded and intact samples was ≥ 0.75, the absolute median abundance ratio was greater than the standard deviation of abundance ratios, and the protein was detected in paired samples from at least 5 of 11 patients post-QC.

### RNA-seq (discovery cohort)

Multiplexed libraries were sequenced on the Illumina HiSeq 2000 (75 bp paired-end read length) and individual indexed library BAM files were produced. Reads that passed quality control (QC) were re-aligned using tophat2^10^, and reads aligning to each gene in each sample were counted using the HTSeq package^11^. Absolute transcript abundance was calculated using the FPKM (fragments per kilobase of transcript per million fragments mapped) measure across 15,418 genes post-QC. We used edgeR v3.0^12^ to identify differentially expressed genes, applying a generalized linear model for tissue status (degraded or intact) with individual ID as covariate, and defined significance at 5% false-discovery rate (FDR).

### Methylation (discovery cohort)

Methylation was assayed using the Illumina 450k BeadChip. Data were parsed and QCed using ChAMP^13^; the probe beta values were quantile-normalized using the ‘dasen’ method from the wateRmelon package^14^. Differential methylation on 425,694 probes post-QC was tested using the CpGassoc package^15^, applying a linear model for tissue status at each probe, with individual ID as covariate.

Following previous analyses^16^, differentially methylated regions (DMRs) were defined as regions of at least 3 probes with FDR ≤ 5% (DMPs) and no more than 3 non-significant probes, with no more than 1 kb between adjacent probes. We used bedtools^17^ to identify genes overlapping each DMR, extending gene boundaries to include likely promoter regions.

For a promoter-level analysis, we used the probe annotations from ChAMP to assign promoter probes to each gene. After computing the mean beta value in the promoter of each gene in each sample, we used a paired t-test to identify genes with differential promoter-region methylation between degraded and intact samples. Significance was set at 5% FDR.

### Replication of gene expression and methylation changes

Gene expression and DNA methylation in both replication cohorts were measured using the same procedure as for the discovery data. We considered the expression of 14,762 genes that passed QC across all datasets; this included 332 of 349 genes with FDR ≤ 5% in the knee discovery data.

We considered 416,437 probes that passed QC across all datasets; this included 9,723 of 9,867 probes with FDR ≤ 5% in the knee discovery data (“DMPs”).

#### In silico replication

We carried out an additional replication analysis for gene expression changes using an available published dataset from the ongoing Research Arthritis and Articular Cartilage (RAAK) study^18^. This dataset comprised a comparison of affected and macroscopically preserved OA cartilage from 33 patients undergoing total joint replacement surgery (22 with hip OA, 11 with knee OA), measured using the Illumina HumanHT-12 v3 microarrays.

### Gene set analyses

We tested whether particular biological gene sets were enriched among significant genes from each of the RNA-seq, methylation, and proteomics datasets. We used KEGG^19^, Reactome^20^, and, separately, Gene Ontology (GO) annotations^21^. Genes annotated to the same term were treated as a “pathway”. Enrichment was assessed using a 1-sided hypergeometric test, with significance at 5% FDR. Empirical p-values for the enrichments were obtained from randomisations.

For each gene set, we asked whether the association across the three –omics datasets (calculated as geometric mean of the *p*-values) was higher than expected by chance, obtaining empirical *p*-values from 100,000 randomisations.

### Ethical approval information

The study was approved by Oxford NHS REC C (10/H0606/20). Tissue samples were collected under National Research Ethics approval reference 15/SC/0132, South Yorkshire and North Derbyshire Musculoskeletal Biobank, University of Sheffield; and under National Research Ethics approval reference 11/EE/0011, Cambridge Biomedical Research Centre Human Research Tissue Bank, Cambridge University Hospitals, UK. All participants provided written informed consent. All methods were carried out in accordance with the relevant guidelines and regulations.

### Data availability

All genome-wide summary statistics generated in the discovery component of this study are available as Supplementary Tables. All discovery RNA sequencing data are available in the EGA repository, https://www.ebi.ac.uk/ega/studies/EGAS00001001203. All discovery methylation data are available in the EGA repository, https://www.ebi.ac.uk/ega/studies/EGAS00001001213. The discovery proteomics data has been deposited in the PRIDE archive under study ID PXD002014 [publication in process].

## Results

We compared the degraded and intact tissue across patient-matched samples.

We identified 209 proteins with evidence of differential abundance (Supplementary Table S1): 90 at higher and 119 at lower abundance in the degraded samples. We confirmed the quality of the data using an orthogonal label-free approach and, separately, Western blotting (see Supplementary Note).

We identified 349 differentially expressed genes: 296 genes showed higher and 53 lower expression levels in the degraded samples (Supplementary Table S2). One of the most strongly down-regulated genes in degraded cartilage was *CHRDL2*. The corresponding protein was also found at lower abundance in degraded cartilage and we confirmed its expression in cartilage by immunohistochemistry (Supplementary Fig. S2). *CHRDL2* is a bone morphogenetic protein (*BMP*) inhibitor that has been reported to be lost from chondrocytes of the superficial zone and shifted to the middle zone in OA cartilage in a targeted study^22^.

We identified 9,896 differentially methylated probes (DMPs) (Supplementary Table S3), and 271 differentially methylated regions (DMRs) composed of multiple differentially methylated CpG sites, overlapping 296 unique genes (Supplementary Table S4).

### Integration across multiple –omics levels

#### Methylation, RNA sequencing, and proteomics

**Figure 2:**
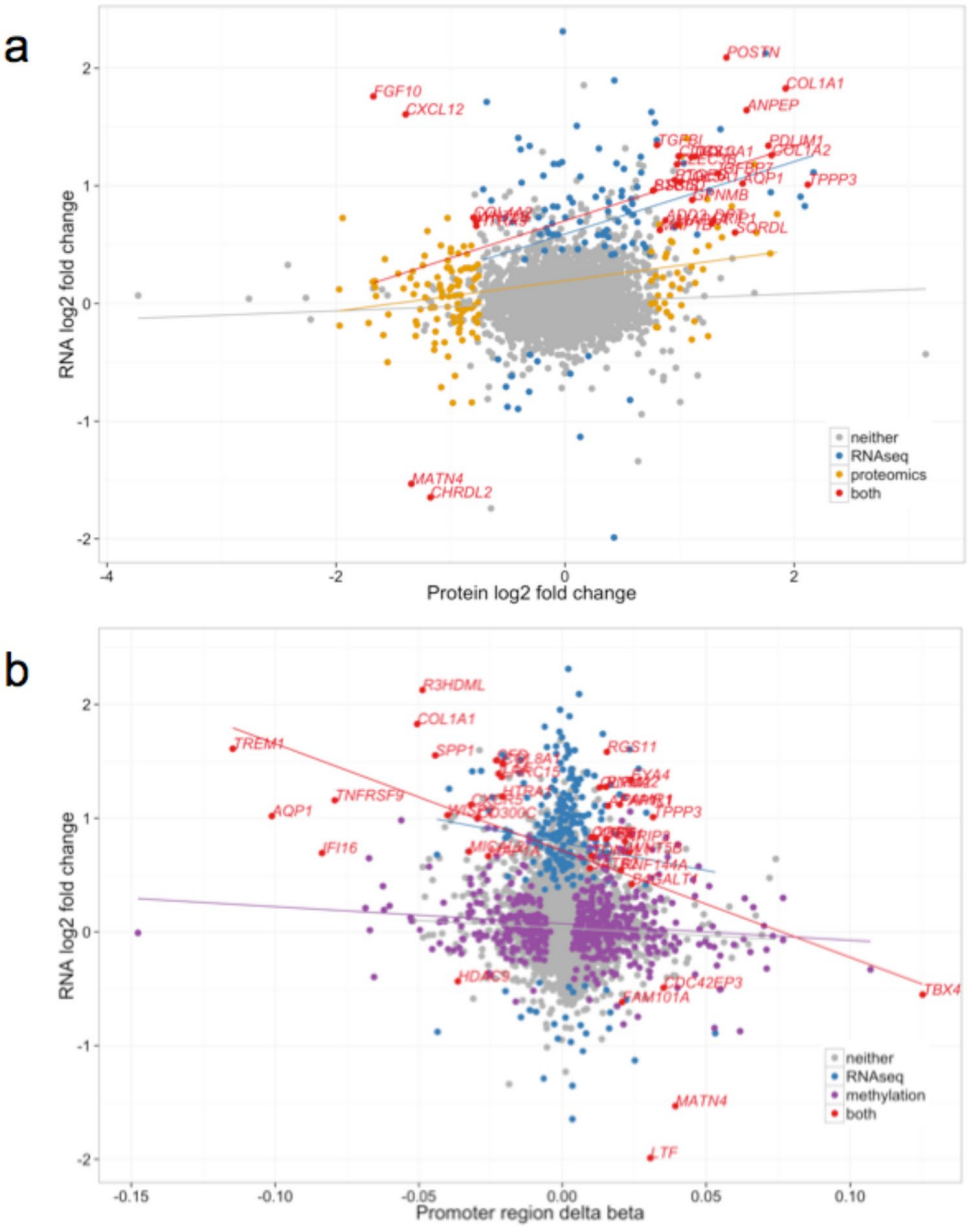
Comparison of changes identified in the –omics experiments. (**a**) Comparison of the log-fold-changes between all genes identified in both the proteomics and RNA-seq experiments. Each gene is represented as a single point, and the colour corresponds to whether the gene is identified as differentially expressed using edgeR in the RNA-seq or proteomics experiments, or both. The trend lines are derived from a linear regression in each subset. Positive fold changes indicate increased expression in degraded samples. (**b**) Comparison of RNA-seq log-fold-change and mean promoter region methylation change. The trend lines are derived from a linear regression in each subset. Genes are coloured according to the results of the RNA-seq and the promoter-region analyses analogously to Fig. 2a.

We identified 49 genes with evidence of differential regulation from at least two of the –omics analyses (Supplementary Table S5). Three genes demonstrated significant evidence for involvement in OA progression across all three levels: *AQP1*, *COL1A1* and *CLEC3B* (Fig. 1b). All three genes were up-regulated in degraded tissue in both the RNA-seq and proteomics analyses (Fig. 2a). *AQP1* and *COL1A1* showed a consistent decrease in methylation of all CpG probes in their associated DMRs (commensurate with an increase in transcription), while the DMR associated with *CLEC3B* showed evidence of increased methylation. Using IHC we independently confirmed the presence of *AQP1*, *COL1A1* and *CLEC3B* in articular cartilage chondrocytes (Supplementary Fig. S2). We also replicated the direction of gene expression change for all three genes in independent data (see below and Supplementary Table S6).

Of the 49 genes with evidence of differential regulation on at least two molecular levels, 33 add substantive evidence to genes previously reported and 16 genes (33%) have not previously been implicated in OA (Supplementary Table S5).

### Proteomics and RNA sequencing

Among the 209 proteins with evidence of differential abundance in the proteomics data, 31 were also differentially expressed between degraded and intact samples at the RNA level (hypergeometric p = 5.3 × 10^−7^, Fig. 1b). Twenty-six of these 31 genes showed concordant directions of changes at both the protein and RNA level (binomial p = 0.0002). Five genes were over-expressed at the RNA level and less abundant at the protein level in the degraded tissue (*COL4A2*, *CXCL12*, *FGF10*, *HTRA3* and *WNT5B*); all five are annotated as secreted proteins in the Human Protein Atlas^23^ (see Supplementary Note).

Using all samples irrespective of tissue status, we found a significant correlation between gene expression levels and protein abundance (Spearman’s rho = 0.29, p < 2.2 × 10^−16^, Supplementary Note and Supplementary Fig. S3). Considering the RNA and protein changes in degraded compared to intact samples (Fig. 2a), we also identified a significant positive correlation (Pearson’s r = 0.17, p < 2.2 × 10^−16^). This correlation became substantially higher when we only considered the 31 genes that were differentially expressed in both datasets (Pearson’s r = 0.43, p = 0.01).

### Methylation and RNA sequencing

Sixteen of the genes overlapping a DMR were also differentially transcribed (Fig. 1b). In the direct comparison of methylation and gene expression using all samples irrespective of tissue status, we found the expected negative correlation between promoter region methylation and gene expression (Spearman’s rho = −0.43, p < 2.2 × 10^−16^, Supplementary Fig. S3). Based on the comparison of intact to degraded cartilage, the log-fold-changes in RNA expression and the differences in mean promoter region methylation values demonstrated a small but highly significant correlation (Pearson’s r = −0.08, p < 2.2 × 10^−16^, Fig. 2b, Supplementary Table S7). The correlation became substantially higher when we considered the 39 genes with significant differences at both the promoter methylation and transcription levels (Pearson’s r = −0.48, p = 0.002).

### Replication of gene expression and methylation changes

**Figure 3:**
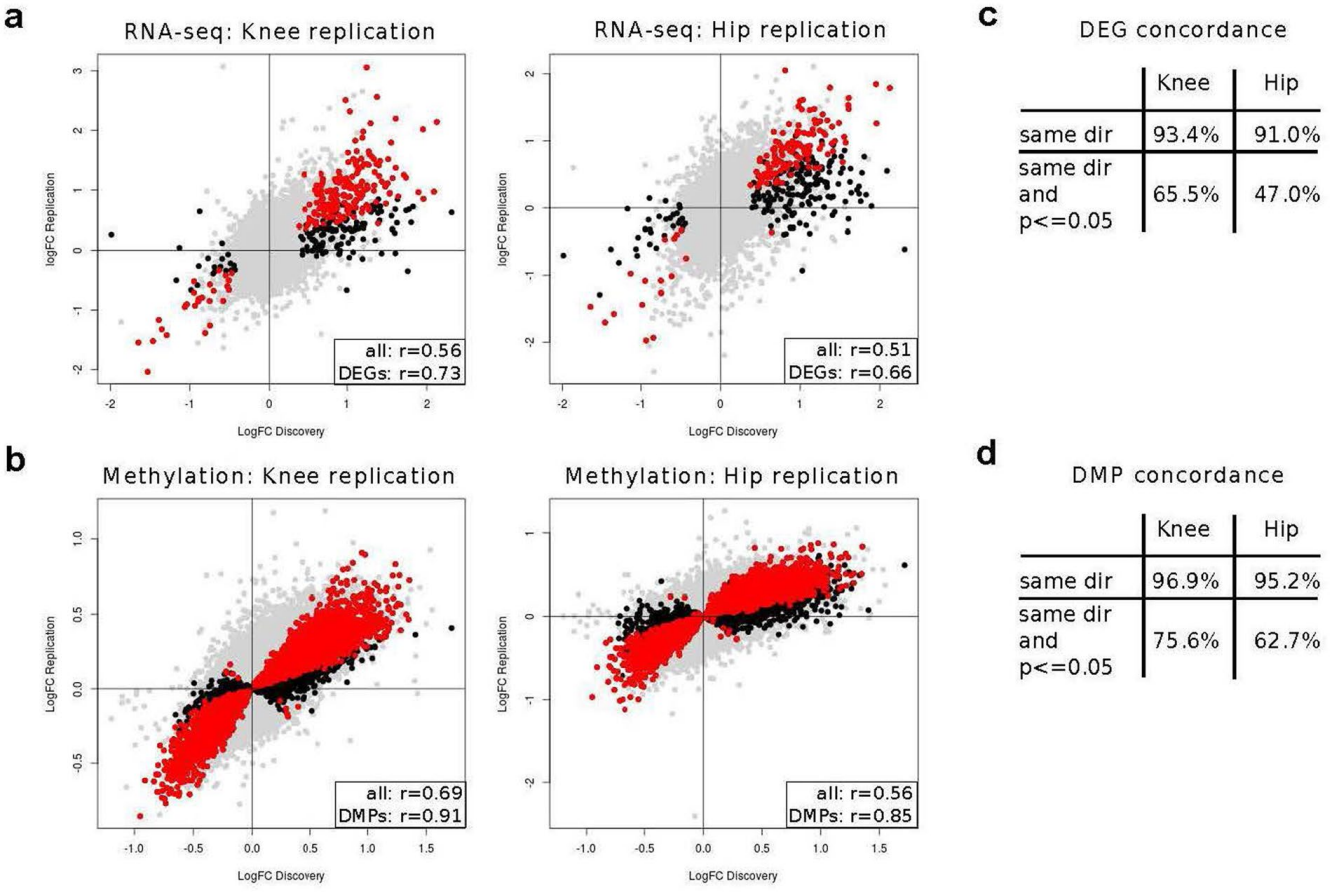
Replication of gene expression and methylation changes. (**a**,**b**) Replication of gene expression changes (**a**) and CpG methylation changes (**b**) in independent datasets of samples from patients with knee (left) and hip (right) OA. Differentially expressed genes (**a**, DEGs) and differentially methylated probes (**b**, DMPs) from the discovery data are marked in black; DEGs and DMPs that additionally show nominal significance in the replication data are marked in red. Inset: correlation between log-fold-changes in discovery and replication data. All: all 14,762 genes (**a**) or 416,437 probes (**b**) that passed QC in the knee discovery, knee replication, and hip replication data. DEGs: 332 of the 349 genes with FDR ≤ 5% in the knee discovery data that also pass QC in both replication datasets; DMPs: 9,723 of the 9,867 probes with FDR ≤ 5% in the knee discovery data that also pass QC in both replication datasets. All correlation values shown have p < 10^−15^. (**c**,**d**) Directional concordance of changes between discovery and replication for the differentially expressed genes (**c**, DEGs) and differentially methylated probes (**d**, DMPs) from the discovery data. Same dir: proportion of DEGs or DMPs with same direction of change in the replication data. Same dir and p ≤ 0.05: among DEGs or DMPs with same direction of change in the replication data, proportion of those that reach nominal significance in the replication dataset.

We found good correlation between the global gene expression log-fold changes and the methylation log-fold changes estimated in the discovery and in the knee and, separately, hip replication data (Fig. 3a,b, Pearson r 0.5–0.9, p < 10^−15^) and excellent replication rates for differentially expressed genes and probes with differential methylation (Fig. 3c,d, over 90% have same direction of change in replication data and at least 47% reach nominal significance, Supplementary Table S8).

We specifically considered the 49 genes with evidence from at least two –omics levels. Of these, 47 had gene expression data in the discovery and both replication datasets; the direction of change replicated at nominal significance for 36 genes in the knee and for 26 genes in the hip replication data (Supplementary Table S6). Notably, the direction of change replicated in at least one of the knee and hip replication datasets at nominal significance for 13 of the 16 genes that have not previously been associated with OA (Supplementary Table S6).

We additionally pursued replication in a further independent, published microarray gene expression dataset from the RAAK study^18^. This study used pooled knee and hip samples, as they found good agreement between the pooled and joint-stratified analyses^18^; to increase power, we also used the pooled data as *in silico* replication. Despite the difference in genomics technology (RNA-seq in discovery, microarray in RAAK), we again found good agreement of the log-fold-changes for the differentially expressed genes from the discovery data (over 60% have same direction of effect, Pearson r in 0.24–0.43 at p ≤ 0.003, see Supplementary Note).

### Pathways involved in OA progression

**Figure 4:**
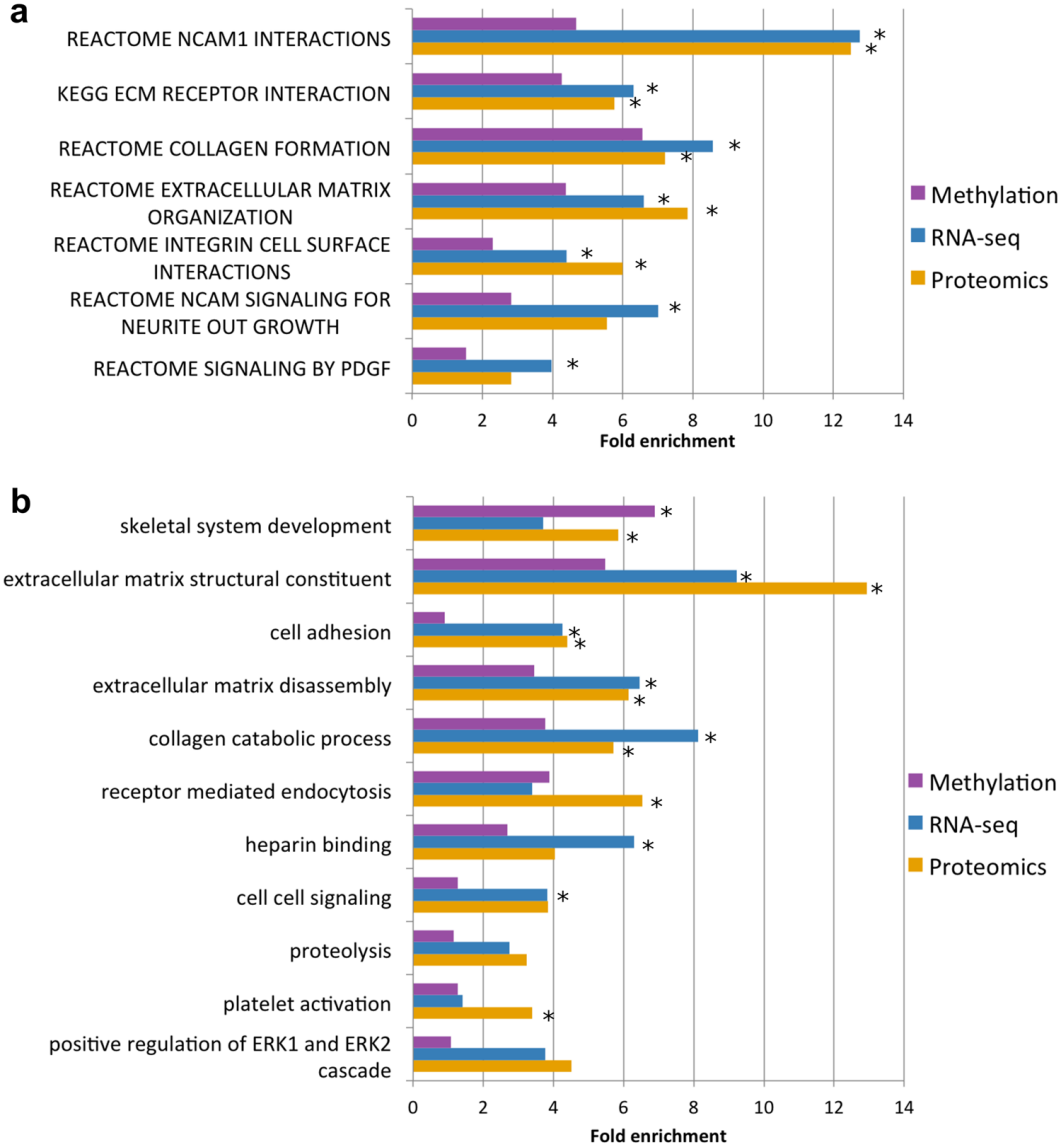
Significantly enriched gene sets in the integrative analysis. Only gene sets that contain at least 5 genes from 2 experiments are shown. Asterisks indicate significant (5% FDR) enrichment for each individual gene-set in an individual –omics experiment. (**a**) Fold enrichment for KEGG & Reactome pathways significant at 5% FDR in the integrative analysis. (**b**) Fold enrichment for GO terms significant at 5% FDR in the integrative analysis.

We identified biological processes with consistent evidence of involvement in OA progression at multiple levels (Fig. 4, Supplementary Fig. S4, Supplementary Tables S9–S10). A strong theme is cartilage matrix regulation, degeneration, and disassembly.

Positive regulation of ERK1/2 cascade, heparin-binding and platelet activation were also enriched at multiple molecular levels and are interconnected through common genes (Supplementary Note). We also found enrichment of genes involved in the regulation of angiogenesis at multiple levels, implicating pathways involved in blood vessel and nervous system formation (NCAM signaling for neurite outgrowth and PDGF signaling). The growth of blood vessels and nerves are closely linked processes that share regulatory mechanisms, including the ERK cascade and heparin-binding proteins^24^. Indeed, histological examination of the samples we investigated showed greater blood vessel ingrowth in tissues with more advanced OA (Supplementary Fig. S1).

### Drug repurposing potential

To identify existing drugs that could be repurposed for OA, we searched Drugbank^25^ for the 49 genes differentially regulated across at least two –omics levels. We identified ten agents with investigational or established actions on nine of the dysregulated proteins, which already have Food and Drug Administration Marketing Authorization for use in humans (Table 1). Among them are non-steroidal anti-inflammatory agents active against prostacyclin synthase (NSAIDs), which already have marketing authorisation for the symptomatic treatment of OA. Our search also identified phylloquinone (vitamin K_1_), an agonist of osteocalcin (*BGLAP* gene). Periostin (*POSTN* gene), a protein with elevated expression in OA in this study and others^26–28^, is a vitamin K-dependent protein that induces cartilage degeneration^29^. A recent study has associated sub-clinical vitamin K deficiency with knee OA incidence^30^, adding evidence that this compound could be a disease-modifying agent in OA.

**Table 1:** Results of Drugbank^25^ (www.drugbank.ca) search for therapeutic compounds with current FDA marketing authorisation for a clinical indication and a potential role in OA treatment.

Gene symbol	Protein	Compound	FDA marketing status	Target specific mechanism	Drug mechanism of action	Reference
*ANPEP*	Aminopeptidase N	Ezetimibe	Prescription	binds protein	Anti-hyperlipidemic medication. Used to lower cholesterol absorption in the small intestine.	Kramer *et al*. (2005) PMID: 15494415
*ANPEP*	Aminopeptidase N	Icatibant	Prescription	inhibits protein	Synthetic peptidomimetic drug consisting of ten amino acids, acts as a specific antagonist of bradykinin B2 receptors. Used in symptomatic treatment of acute attacks of hereditary angioedema in adults with C1-esterase-inhibitor deficiency.	Bawolak *et al*. (2006) PMID: 17026984
*AQP1*	Aquaporin-1	Acetazolamide	Prescription	inhibits protein	Carbonic anhydrase inhibitor diuretic agent. Used for the medical treatment of glaucoma, epileptic seizure, idiopathic intracranial hypertension, altitude sickness, cystinuria, periodic paralysis, central sleep apnea, and dural ectasia.	Xiang *et al*. (2004) PMID: 15169637
*BGLAP*	Osteocalcin	Phylloquinone (vitamin K_1_)	NA (vitamin)	agonist, carboxylates protein	Fat-soluble vitamin necessary for post-translational modification of certain proteins, mostly required for blood coagulation.	Schurgers *et al*. (2001) PMID: 11374034
*CLEC3B*	Tetranectin	Tenecteplase	Prescription	binds protein	Tissue plasminogen activator (tPA). Used as a thrombollytic agent.	Westergaard *et al*.^46^ PMID: 12694198
*CXCL12*	Stromal cell-derived factor 1	Tinzaparin	Prescription	binds protein	Low molecular weight heparin (LMWH). Used in the treatment and prophylaxis of venous thrombo-embolism.	Koo *et al*. (2008) PMID: 18991783
*FGFR2*	Fibroblast growth factor receptor 2	Palifermin	Prescription	binds protein	Recombinant human keratinocyte growth factor (KGF). Used to treat oral mucositis in patients undergoing cancer chemotherapy.	Beaven *et al*. (2007) PMID: 17728847
*MAP1A*	Microtubule-associated protein 1 A	Estramustine	Prescription	disrupts protein	A nitrogen mustard linked to estradiol. Used in palliative care of prostatic neoplasms.	Stearns *et al*. (1991) PMID: 1647395
*PTGIS*	Prostacyclin synthase	Non-steroidal anti-inflammatory agents	Prescription	inhibits protein	COX1 and COX2 inhibitors used in the symptomatic treatment of OA.	Reed *et al*. (1985) PMID: 3917545
*S100A4*	Protein S100-A4	Trifluoperazine	Prescription	inhibits protein function	A phenothiazine with actions similar to chlorpromazine. Used as an antipsychotic and an antiemetic.	Malashkevish *et al*. (2010) PMID: 20421509

The mechanisms of action and references are taken from Drugbank.

## Discussion

Characterisation of the molecular landscape of OA is now increasingly feasible and catalyzed by technological advances in functional genomics, and the accessibility of the relevant tissue at joint replacement surgery. Indeed, the field of functional genomics in OA is now budding^31^. Previous studies of osteoarthritis have investigated methylation^32, 33^, transcription^18, 34–37^, or protein expression^38, 39^ separately, or a combination of up to two of these –omics assays^16, 40, 41^, some with the addition of genetic data^42^. By contrast, this study provides a systematic analysis of biological changes involved in OA across genome-wide methylation, gene and protein expression levels.

We identify 49 genes with evidence of changes between intact and degraded cartilage on multiple molecular levels, including some novel molecular players in OA. We provide robustly-replicating evidence with convergence of all three –omics levels of the involvement of *AQP1*, *COL1A1*, and *CLEC3B* in disease progression.

*AQP1* encodes aquaporin-1, a protein that facilitates water transport across biological membranes. Chondrocyte swelling and increased cartilage hydration has been suggested as an important mechanism in OA^43^. *AQP1* has been found to be over-expressed in the meniscus of a rat model of knee OA^44^ and in degraded compared to intact articular cartilage in human patients with knee OA^45^. *CLEC3B* encodes tetranectin, which binds human tissue plasminogen activator (tPA)^46^. *CLEC3B* has previously been found to be up-regulated in OA^34, 47^. *COL1A1* is one of several collagens that were differentially abundant at both the RNA and protein levels (see Supplementary Table S5). Collagens are the main structural components of cartilage and collagen dysregulation plays an important role in OA^48, 49^. A recent study identified up-regulation of *COL1A1* in synovium from humans with end-stage OA, in the synovium of mice with induced OA and in human fibroblasts stimulated with TGF-β^50^. Moreover, the products of *AQP1* and *CLEC3B* are targeted by existing drugs approved for human use: aquaporin-1 is inhibited by Acetazolamide, a carbonic anhydrase inhibitor diuretic agent, and tetranectin is bound by Tenecteplase, a thromobolytic agent (Table 1).

We further identify previously unreported genes with concordant molecular changes in the degraded tissue on multiple levels, and with robust replication in independent datasets. *MAP1A* and *MAP1B* were both significantly up-regulated in degraded cartilage at the RNA and protein levels (Fig. 2a). These proteins are mainly expressed in the brain and involved in regulating the neural cytoskeleton^51^. Cytoskeletal regulation is thought to be an important process in OA^52^ and recently these proteins were implicated in bone formation^53^. *PXDN* was up-regulated at the RNA level, confirmed by replication, and overlapped two hypo-methylated DMRs. *PXDN* encodes peroxidasin, which is secreted into the extracellular matrix and catalyses collagen IV cross-linking^54^. The other replicated genes not previously implicated in OA (Supplementary Table S6) have relatively little characterisation.

*ANPEP* (aminopeptidase E) is a broad specificity aminopeptidase that has previously been detected in the synovial fluid of OA patients^55^ and therefore has potential as a novel OA biomarker. *CHRDL2* is a bone morphogenetic protein (*BMP*) inhibitor that has been reported to be lost from chondrocytes of the superficial zone and shifted to the middle zone in OA cartilage in a targeted study^22^. *WNT5B*, a ligand for frizzled receptors in the WNT signaling pathway, has been found to be differentially transcribed in osteoarthritic bone, consistent with current understanding that OA is a disease involving both cartilage and bone^56^.

We identify extracellular matrix organization, collagen catabolism and angiogenesis as biological pathways that have significant association with disease progression, in agreement with the consensus findings of previous methylation and gene expression studies of OA^7^. These results corroborate that increased ECM turnover is a crucial component in OA pathogenesis. We found suggestive evidence of a link between some of these pathways and genetic OA risk loci (see Supplementary Note, Supplementary Table S11). These signals would not have been identified directly from GWAS data, highlighting the importance of synthesizing information from multiple molecular levels to obtain a more powerful integrated view. Follow-up mechanistic studies will be required before causal relationships between the identified pathways and OA progression can be established. In the chondrocyte, we found little evidence of differential inflammatory pathway activity between the intact and degraded samples^57, 58^. This is not surprising, as all of the patients studied here had a diagnosis of OA and clinically advanced disease in at least one location within the joint. Inflammatory mediators are soluble factors present throughout the joint, to which both the healthy and the diseased chondrocyte populations are potentially exposed, and for which the regulatory molecules may differ compared to the articular cartilage tissue studied here.

Based on our discovery data, we estimate that ~95% of significantly different genes identified in this study are true positives (Supplementary Fig. S5). The replication data confirm the high true positive rate and yield independent strong evidence for multiple novel OA genes. This shows that the gene expression and methylation changes identified are robust. Within the constraints of this study, the changes between intact and degraded OA cartilage are largely joint-independent. Further work will be needed to narrow down which individual gene-level differences between intact and degraded cartilage are joint-independent and which are specific to a joint. This work is complementary to previous studies which examined differences in lesion-distant cartilage between joints^59^ or by pooling intact and degraded cartilage^16^, as neither of these studies examined differences between intact and degraded cartilage, and whether such differences are joint-independent.

Notably, this study is a proof-of-concept for integrative deep molecular phenotyping across methylation, gene expression, and protein abundance. As such, it was not powered to provide an exhaustive list of molecular targets and pathways. Indeed, we estimate that only ~10% of the true differentially expressed genes are statistically significant in this study (Supplementary Fig. S5). The sample size could also affect the degree of overlap and agreement between the methylation, gene expression, and protein abundance (this overlap could also be affected by difficulties in assigning the effects of methylation changes to genes: for example, it is possible that the expression of a gene is affected by methylation changes in a distal enhancer, or that a given gene contains an enhancer region for a different gene, and thus methylation of the first gene also affects the expression of the second). Larger sample sizes will be required for a more powerful characterisation of the molecular changes occurring with disease progression. Moreover, investigations of further OA-relevant cell types (including synoviocytes and adipocytes) will be necessary to identify disease-related changes in other tissues, and the biological mechanisms specific to such tissues.

In summary, the integrative functional genomics approach undertaken here has identified biological changes in disease-relevant tissues, highlighting three genes and several pathways that are involved on all three levels examined. Moreover, the approach identified nine genes with changes on multiple molecular levels that are already targeted by drugs approved for human use, highlighting the potential of discovering targets for intervention. These drugs have established safety profiles and pharmacokinetic data for use in humans, which would shorten the investigative pipeline to clinical use in OA. These agents cover a broad range of mechanisms of action and represent novel investigational targets for ‘first in disease’ studies of OA progression. Further studies will be necessary to comprehensively characterize the molecular signatures of OA.

## Electronic supplementary material

Supplementary Information

Supplementary Table 1

Supplementary Table 2

Supplementary Table 3

Supplementary Table 4

Supplementary Table 5

Supplementary Table 6

Supplementary Table 7

Supplementary Table 8

Supplementary Table 9

Supplementary Table 10

Supplementary Table 11

Supplementary Table 12

Supplementary Table 14
