# Supplementary material for: Integrative epigenomics, transcriptomics and proteomics of patient chondrocytes reveal genes and pathways involved in osteoarthritis
Source: Sci Rep. 2017 Aug 21;7:8935. doi: 10.1038/s41598-017-09335-6 (PMC5566454; doi:10.1038/s41598-017-09335-6)
Supplement: Supplementary file 1 — Supplementary Information [file 41598_2017_9335_MOESM1_ESM.pdf]

Integrative epigenomics, transcriptomics and proteomics of patient chondrocytes reveal genes and pathways involved in osteoarthritis: online supplementary material

AUTHORS

Julia Steinberg[1,#], Graham R. S. Ritchie[1,2,3,4,#], Theodoros I. Roumeliotis[1], Raveen L. Jayasuriya[5], Matthew J Clark[5], Roger A. Brooks[6], Abbie L. A. Binch[7], Karan M. Shah[5], Rachael Coyle[1], Mercedes Pardo[1], Christine L. Le Maitre[7], Yolande F. M. Ramos[8], Rob G. H. H. Nelissen[9], Ingrid Meulenbelt[8], Andrew W. McCaskie[6], Jyoti S. Choudhary[1], J. Mark Wilkinson[5*#], Eleftheria Zeggini[1*#]

SUPPLEMENTARY NOTE

Quantitative proteomics

For two representative patients we also used an orthogonal label-free approach that confirmed the protein quantification data (Supplementary Fig. S6).
One of the most strongly down-regulated proteins is hyaluronan and proteoglycan link protein 1 (*HAPLN1*), which binds hyaluronic acid in the extracellular matrix. *HAPLN1* was found to be more abundantly released to the culture media by OA cartilage explants compared to healthy control tissue [1]. Intra-articular injection of hyaluronic acid is one of the few current targeted treatments for OA pain [2]. Based on UniProt annotation [3] we found a number of proteoglycans, cartilage and chondrocyte function-related proteins as well as basement membrane proteins consistently at lower abundances in the degraded samples (Supplementary Table S12). This is potentially a reflection of the increased cartilage catabolism that occurs in OA. As is the case for *HAPLN1*, several of these are found increasingly in the media released by OA tissue or synovial fluid from OA patients [1,4].

Western blotting

In order to validate the quantitative proteomic mass spectrometry results, we selected several proteins with consistent changes and analysed these in paired degraded/intact cartilage samples by quantitative Western blotting: ANPEP, AQP1, TGFB1, and WNT5B. ANPEP, AQP1, and TGFB1 levels were found to be reproducibly increased in degraded cartilage samples compared to their intact counterparts (Supplementary Table S13, Supplementary Fig. S7). All three were also significantly differentially expressed at the RNA level.
WNT5B levels were increased in degraded cartilage samples compared to intact cartilage samples in half of the patients analysed, with little variation in the other pairs (Supplementary Fig. S7, Supplementary Table S13).
Overall, there is a good agreement between the western blot and spectrometry analysis, with the exception of WNT5B (Supplementary Fig. S8; the latter partially due to weak signal in half the samples pairs, ids 49,51,54,55).
TGFBI/BGH3 is an adhesion protein induced by TGF-β that binds to ECM proteins, integrins and to periostin, another up-regulated protein in OA which promotes cartilage degeneration through WNT signalling [5,6]. TGFBI protein is released by OA tissue explants [1] and is also found in the synovial fluid of OA patients, but is more abundant in that of rheumatoid arthritis patients [4]. *ANPEP*, *AQP1*, and *WNT5B* are discussed in the main manuscript.

Down-regulated proteins

We found that the RNA levels of the genes encoding several proteins down-regulated in degraded cartilage samples (Supplementary Table S12) were not found to be significantly changed, with the exception of *MATN4* which also showed decreased RNA levels. The molecular profiles of these genes may imply post-translational regulation of protein abundance. A protein with unknown function, namely C17orf58, was consistent with the above group suggesting a novel target for functional annotation.

Differential methylation

Among the sixteen genes found in DMRs and among the genes with significant changes from the RNA sequencing data, we found two members of the ADAMTS family: *ADAMTS2* and *ADAMTS4*. Both genes are up-regulated in degraded cartilage samples based on the RNA sequencing data and were consistently associated with hypo-methylated DMRs. We also identified several other members of this family to be either transcriptionally up-regulated (*ADAMTS12* and *ADAMTS14*) or associated with a hypo-methylated DMR (*ADAMTS17*).

These genes encode peptidases that catabolise components of the extracellular matrix, including aggrecan, which was the single most down-regulated protein in degraded cartilage samples. Previous studies have implicated this gene family in OA [7] and have suggested that *ADAMTS4*-mediated aggrecan degradation may be an important process [8]. Accordingly, aggrecan is more abundant in OA synovial fluid [4].

Integration across multiple –omics levels

Proteomics and RNA sequencing

Five genes were over-expressed at the RNA level and less abundant at the protein level in the degraded tissue (*COL4A2*, *CXCL12*, *FGF10*, *HTRA3* and *WNT5B*); all five are annotated as secreted proteins in the Human Protein Atlas [9], all five proteins encoded by these genes are annotated as predicted secreted proteins. In agreement with this, several collagens are more abundantly released into the culture media from diseased tissue than from healthy tissue [1].

We computed the global correlation in all samples irrespective of tissue status, comparing the RNA fragments per kilobase of transcript per million fragments mapped (FPKM) [10] to normalized peptide spectral counts (Supplementary Fig. S3) and found a significant positive correlation (Spearman's rho=0.29, $p<2.2x10^{-16}$) between RNA expression levels and protein abundance. To establish if there were also concordant differences in RNA and protein abundance between intact and degraded tissue, we computed the correlations between RNA and protein changes in degraded compared to intact samples (Figure 2a), and identified a significant positive correlation (Pearson's r=0.17, $p<2.2x10^{-16}$). The magnitude of correlation became substantially stronger when we only considered the 31 genes that were expressed differentially in both datasets (Pearson's r=0.43, p=0.01).

Methylation and RNA sequencing

Sixteen of the genes with an associated DMR were also differentially transcribed (Figure 1b). For the direct comparison of methylation and gene expression in the following, we used the aggregate methylation status of promoter region CpG probes with transcription levels in all samples irrespective of intact/degraded status (Supplementary Table S7). We found the expected negative correlation between promoter region methylation and gene expression (Spearman's rho=-0.43, $p<2.2x10^{-16}$, Supplementary Fig. S3). Based on the comparison of intact to degraded cartilage, the log-fold-changes in RNA expression and the differences in mean promoter region methylation values demonstrated a small but highly significant correlation (Pearson's r=-0.08, $p<2.2x10^{-16}$, Figure 2b). The correlation became substantially higher when we considered the 39 genes with significant differences at both the promoter methylome and transciptome levels (Pearson's r=-0.48, p=0.002).

Replication of gene expression changes

We assayed gene expression in degraded and intact cartilage samples from two independent cohorts: a set of 17 patients with knee OA and a set of 9 patients with hip OA, using the same approach as for the discovery data. After quality control, we retained 14,762 genes common to the discovery and both replication datasets, including 332 of 349 genes with FDR≤5% in the discovery data. We found excellent concordance in the direction of change for the genes with FDR≤5% in the discovery data: 93.4% of genes showed the same direction of effect in the knee replication data and 91.0% of genes showed the same direction of effect in the hip replication data (Figure 3a; both binomial $p<10^{-15}$). Of the genes with concordant effect between the discovery and replication data, 65.5% reached nominal statistical significance in the knee replication data and 47% in the hip replication data (Supplementary Table S8; both binomial $p<10^{-15}$).

In addition, we found good correlation for the estimates of the log-fold-changes across all 14,762 genes between the knee discovery and the replication data: r=0.56 for knee replication data and r=0.51 for hip replication data (both $p<10^{-15}$). These correlations are higher for the 332 genes with FDR≤5% in the discovery gene expression data (r=0.73 for knee, r=0.66 for hip replication data, both $p<10^{-15}$). This shows that the gene expression changes identified in this study are robust and largely joint-independent.

We specifically considered the 49 genes with evidence from at least two –omics levels. Of these, 47 had gene expression data in the discovery and both replication datasets; 36 genes had nominally significant differential gene expression in the same direction in the knee replication data, and 26 genes in the hip replication data (Supplementary Table S6). This included *ANPEP*, for which we have used Western blotting to confirm protein changes (Supplementary Fig. S7), and *CHRDL2*, for which we used immunohistochemistry to confirm presence of the protein (Supplementary Fig. S2). Notably, the direction of change replicates in at least one of the knee and hip replication datasets at nominal significance for 13 of the 16 genes that have not previously been associated with OA (Supplementary Table S6).

We additionally pursued replication in an independent published microarray gene expression dataset of degraded and intact cartilage from the RAAK study, including 22 individuals with hip OA and 11 individuals with knee OA [11]. Of the 349 genes with FDR≤5% in the discovery dataset, 154 genes had expression measurements in the RAAK knee and hip replication datasets. We found highly significant agreement between the discovery and RAAK data: 83.8% of the genes showed the same direction of effect in the knee RAAK data (binomial one-sided $p<10^{-15}$) and 69.5% of genes showed the same direction of effect in the hip RAAK data (binomial one-sided $p<10^{-6}$). Furthermore, despite the difference in genomics technology (RNA-seq in discovery, microarray in RAAK), we found good concordance for the estimates of the log-fold-changes between the knee discovery and the RAAK replication data: r=0.43 for knee ($p=3.6\times10^{-8}$) and r=0.24 for hip replication data (p=0.003).

Replication of methylation changes

To replicate the methylation results, we assayed DNA methylation in degraded and intact cartilage samples from two independent datasets: a set of 17 patients with knee OA and a set of 8 patients with hip OA, using the same approach as for the discovery data. After quality control, we retained 416,437 probes common to the discovery and both replication data sets, including 9,723 of 9,867 differentially methylated probes (DMPs) with FDR≤5% in the knee discovery data. We found excellent concordance in the direction of change for the DMPs: 96.9% of probes showed the same direction of effect in the knee replication data and 95.2% probes showed the same direction of effect in the hip replication data (Figure 3b; both binomial one-sided $p<10^{-15}$).

Furthermore, we found good correlation for the estimates of the fold-changes across all 416,437 probes between the knee discovery and the replication data: r=0.69 for knee replication data and r=0.56 for hip replication data (both $p<10^{-15}$). The correlation is even higher for the DMPs: r=0.91 for knee, r=0.85 for hip replication data (both $p<10^{-15}$). Similarly to the gene expression data, this shows that the methylation changes estimated in this study are robust and largely joint-independent.

In summary, our combined epigenetic, transcriptomic and proteomic analysis has uncovered a substantial number of robustly replicating genes associated with OA progression, some with known connections to cartilage or bone-related processes, and others with no links

reported to date, bringing new potential insights into the molecular mechanisms of OA pathogenesis.

Pathways involved in OA progression

Positive regulation of ERK1/2 cascade, heparin-binding and platelet activation were also enriched at multiple molecular levels and are interconnected through common genes. Several studies have linked the extracellular signal-regulated kinase (ERK) cascade to OA [12-16]. Heparin-binding growth factors have also been shown to be involved in OA [17-20], some in particular through activation of the ERK signaling pathway. Injection of platelet-rich plasma in OA knees leads to significant clinical improvement [21,22] and there is evidence to suggest that this effect is mediated via the ERK cascade [23]. Our findings provide strong evidence supporting a role for this pathway in OA pathogenesis.

We also found significant enrichment in plasma proteins for both the RNA-seq (hypergeometric $p=6.9x10^{-11}$) and the proteomics experiments ($p=1.8x10^{-5}$). This supports a role for angiogenesis and nerve growth in OA progression [24,25].

Overlap with genes proximal to GWAS signals

We asked whether genes located in loci associated with osteoarthritis in GWAS showed evidence of association in this study. We obtained 19 SNPs from 16 loci that showed genome-wide significant association with knee or hip osteoarthritis [26,27]. For each SNP, we obtained all genes located within 500kb either side of the SNP from Ensembl Biomart (GRCh38). For each of the genes, if available, we list the results from the RNA-seq, methylation, and protein expression datasets.

For 9 signals we identified at least one gene within 500kb around the lead variant (Supplementary Table S14; multiple genes for 3 signals). However, no gene was supported by more than one of the methylation, RNA-seq or proteomics experiments, and none of the genes was highly significant (all with FDR>1.5%).

arcOGEN gene-set association analysis

We performed a gene-set association analysis using the arcOGEN GWAS data [28] to establish if any of the 18 gene sets we highlighted were enriched for genetic loci associated with OA risk, and found 5 gene sets with significant association statistics at a 5% FDR threshold (Supplementary Table S11), including the GO terms "extracellular matrix disassembly" and "collagen catabolic process". When we tested other gene sets of similar size but not highlighted by our –omics analyses, we observed that at least 1 in 10 had empirical *p* values as low as the highlighted gene sets. A direct, hypothesis-free gene-set analysis of the arcOGEN GWAS data analogous to the omics enrichment tests identified very little enrichment.

SUPPLEMENTARY METHODS

Patients recruitment (discovery cohort)

All subjects provided written, informed consent prior to participation in the study. Tissue samples were collected under Human Tissue Authority license 12182, Sheffield Musculoskeletal Biobank, University of Sheffield, UK. All samples were collected from patients undergoing total knee replacement for primary osteoarthritis. The patients comprised 2 women and 10 men, mean age 66 years (range 50-88). Patients with diagnosis other than osteoarthritis were excluded from the study. The study was approved by Oxford NHS REC C (10/H0606/20). Patients with a history of glucocorticoid use (systemic or intra-articular) within the previous 6 months, or use of any other drugs associated with immune modulation were excluded from participation, as were those with any history of fracture, significant knee surgery (apart from meniscectomy), knee infection, or any malignancy within the previous 5 years.

Cartilage tissue was graded using the Mankin Score (0-14) with additional scores for abnormal features (0-4) and cartilage thickness (0-4) based on the OARSI scoring system [29,30]. The total scores were used to determine the overall grade of the cartilage as healthy/low-grade degenerate, referred to as “intact” (median: 4.5; IOR: 3-5.5; n=12), or high-grade degenerate, referred to as “degraded” (median: 14; IOR: 11.75-18; n=12).

Sample processing

Extraction of chondrocytes from osteochondral tissue taken at knee replacement

Osteochondral samples were transported in Dulbecco's modified Eagle's medium (DMEM)/F-12 (1:1) (Life Technologies) supplemented with 2mM glutamine (Life Technologies), 100 U/ml penicillin, 100 µg/ml streptomycin (Life Technologies), 2.5 µg/ml amphotericin B (Sigma-Aldrich) and 50 µg/ml ascorbic acid (Sigma-Aldrich) (serum free media). Half of each sample was taken for chondrocyte extraction and the remaining tissue was fixed in 10% neutral buffered formalin, decalcified in surgipath decalcifier (Leica) and embedded to paraffin wax for histological and immunohistochemical analysis. Chondrocytes were directly extracted from each paired macroscopic intact and degraded OA cartilage sample in order to remove the extracellular matrix allowing a higher yield of cells to be loaded onto the Qiagen column.

Histological examination

Four micron sections of paraffin-embedded cartilage tissue were mounted onto positively charged slides and histologically stained using Haematoxylin and Eosin, Alcian blue, Masson trichrome. Sections were dewaxed in Sub-X, rehydrated in IMS, washed in distilled water, stained in 1% w/v Alcian blue/glacial acetic acid (pH 2.4) for 15 minutes, counter stained in 1% w/v aqueous neutral red for 1 minute or stained with Masson Trichrome (Leica) according to the manufacturer’s instructions. Sections were dehydrated and mounted.

Extraction of DNA, RNA, and protein

Cartilage was removed from the bone, dissected and washed twice in 1xPBS. Tissue was digested in 3 mg/ml collagenase type I (Sigma-Aldrich) in serum free media overnight at 37°C on a flatbed shaker. The resulting cell suspension was passed through a 70 µm cell strainer (Fisher Scientific) and centrifuged at 400g for 10 minutes; the cell pellet was then washed twice in serum free media, followed by centrifugation at 400g for 10 minutes. The resulting cell pellet was resuspended in serum free media. Cells were counted using a haemocytometer and the viability checked using trypan blue exclusion (Invitrogen). The optimal cell number for spin column extraction from cells was between $4x10^6$ and $1x10^7$. Cells were then pelleted and homogenized. DNA, RNA and protein extractions were

performed using the Qiagen AllPrep DNA/RNA/Protein Mini Kit, as per manufacturer's instructions. RNA, DNA and protein were quantitated by picogreen and gel electrophoresis. Samples were frozen at -80 degrees C prior to assays.

Proteomics

Protein Digestion and TMT Labeling

Paired protein samples were obtained from 11 of the 12 patients.

The protein content of each sample was precipitated by the addition of 30 µL TCA 8 M at 4 °C for 30 min. The protein pellets were washed twice with ice cold acetone and finally re-suspended in 40 µL 0.1 M triethylammonium bicarbonate, 0.05% SDS with pulsed probe sonication. Protein concentration was measured with Quick Start Bradford Protein Assay (Bio-Rad) according to manufacturer's instructions. Aliquots containing 30 µg of total protein were prepared for trypsin digestion. Cysteine disulfide bonds were reduced by the addition of 2 µL 50 mM tris-2-carboxymethyl phosphine (TCEP) followed by 1 h incubation in heating block at 60 °C. Cysteine residues were blocked by the addition of 1 µL 200 mM freshly prepared Iodoacetamide (IAA) solution and 30 min incubation at room temperature in dark. Trypsin (Pierce, MS grade) solution was added at a final concentration 70 ng/µL to each sample for overnight digestion. After proteolysis the peptide samples were diluted up to 100 µL with 0.1 M TEAB buffer. A 41 µL volume of anhydrous acetonitrile was added to each TMT 6-plex reagent (Thermo Scientific) vial and after vortex mixing the content of each TMT vial was transferred to each sample tube. Labeling reaction was quenched with 8 µL 5% hydroxylamine for 15 min after 1 h incubation at room temperature. Samples were pooled and the mixture was dried with speedvac concentrator and stored at -20 °C until the high-pH Reverse Phase (RP) fractionation.

Peptide fractionation

Offline peptide fractionation based on high pH Reverse Phase (RP) chromatography was performed using the Waters, XBridge C18 column (2.1 x 150 mm, 3.5 µm, 120 Å) on a Dionex Ultimate 3000 HPLC system equipped with autosampler. Mobile phase (A) was composed of 0.1% ammonium hydroxide and mobile phase (B) was composed of 100% acetonitrile, 0.1% ammonium hydroxide. The TMT labelled peptide mixture was reconstituted in 100 µL mobile phase (A), centrifuged and injected for fractionation. The multi-step gradient elution method at 0.2 mL/min was as follows: for 5 minutes isocratic at 5% (B), for 35 min gradient to 35% (B), gradient to 80% (B) in 5 min, isocratic for 5 minutes and re-equilibration to 5% (B). Signal was recorded at 280 nm and fractions were collected in a time dependent manner every one minute. The collected fractions were dried with SpeedVac concentrator and stored at -20 °C until the LC-MS analysis.

LC-MS Analysis

LC-MS analysis was performed on the Dionex Ultimate 3000 UHPLC system coupled with the high-resolution LTQ Orbitrap Velos mass spectrometer (Thermo Scientific). Each peptide fraction was reconstituted in 40 µL 0.1% formic acid and a volume of 10 µL was loaded to the Acclaim PepMap 100, 100 µm × 2 cm C18, 5 µm, 100 Å trapping column with a user modified injection method at 10 µL/min flow rate. The sample was then subjected to a multi-step gradient elution on the Acclaim PepMap RSLC (75 µm × 50 cm, 2 µm, 100 Å) C18 capillary column (Dionex) retrofitted to an electrospray emitter (New Objective, FS360-20-10-N-20-C12) at 45 °C. Mobile phase (A) was composed of 96% H_2O, 4% DMSO, 0.1% formic acid and mobile phase (B) was composed of 80% acetonitrile, 16% H_2O, 4% DMSO, 0.1% formic acid. The gradient separation method at flow rate 300 nL/min was as follows: for 95 min gradient to 45% B, for 5 min up to 95% B, for 8 min isocratic at 95% B, re-equilibration to 5% B in 2 min, for 10 min isocratic at 5% B.

The ten most abundant multiply charged precursors within 380 -1500 *m/z* were selected with FT mass resolution of 30,000 and isolated for HCD fragmentation with isolation width 1.2 Th. Normalized collision energy was set at 40 and the activation time was 0.1 ms for one microscan. Tandem mass spectra were acquired with FT resolution of 7,500 and targeted precursors were dynamically excluded for further isolation and activation for 40 seconds with 10 ppm mass tolerance. FT max ion time for full MS experiments was set at 200 ms and FT MSn max ion time was set at 100 ms. The AGC target vales were 3×10e6 for full FTMS and 1×10e5 for MSn FTMS. The DMSO signal at m/z 401.922718 was used as a lock mass.

Database Search and Protein Quantification

The acquired mass spectra were submitted to SequestHT search engine implemented on the Proteome Discoverer 1.4 software for protein identification and quantification. The precursor mass tolerance was set at 30 ppm and the fragment ion mass tolerance was set at 0.02 Da. TMT6plex at N-termimus, K and Carbamidomethyl at C were defined as static modifications. Dynamic modifications included oxidation of M and Deamidation of N,Q. Maximum two different dynamic modifications were allowed for each peptide with maximum two repetitions each. Peptide confidence was estimated with the Percolator node. Peptide FDR was set at 0.01 and validation was based on q-value and decoy database search. All spectra were searched against a UniProt fasta file containing 20,190 Human reviewed entries. The Reporter Ion Quantifier node included a custom TMT 6plex Quantification Method with integration window tolerance 20 ppm and integration method the Most Confident Centroid. For each identified protein a normalized spectral count value was calculated for each one of the 6-plex experiments by dividing the number of peptide spectrum matches (PSMs) of each protein with the total number of PSMs. Median normalized spectral counts per protein were computed across the different multiplex experiments.

Western blotting

Sample pairs were adjusted to the same protein concentration. Twenty micrograms of protein per sample were electrophoresed on 4-12% Bis-Tris NuPAGE gels (Life Technologies) and transferred to nitrocellulose membranes. Primary antibodies used were as follows: ANPEP, ab108382; AQP1, ab168387; COL1A, ab14918; TGFB1, ab89062; WNT5B, ab124818 (Abcam); GAPDH, sc-25778 (Santa Cruz Biotechnologies). Chemiluminescence detection was carried out using ECL Prime (GE Healthcare) or ECL Ultra (Lumigen) and ImageQuant LAS1400 (GE Healthcare). Densitometry was performed with ImageQuant Tool Box (GE Healthcare). Intensity values were normalised to GAPDH loading control before ratio calculation.

Label free quantification of representative samples

For a selection of four representative control and disease samples, peptide aliquots of 500ng without TMT labelling were analysed on the Dionex Ultimate 3000 UHPLC system coupled with the Orbitrap Fusion (Thermo Scientific) mass spectrometer for label free quantification and validation. Tandem mass spectra were acquired over duplicate runs of 120 min with a top speed iontrap detection method and dynamic exclusion at 10 sec and MS R=120,000. Database search was performed on Proteome Discoverer 1.4 with the SequestHT engine and normalized spectral counts were computed based on the total number of peptide-spectrum matches attributed to each protein per sample divided by the maximum value along the different samples. With a minimum requirement of at least total 14 spectra per protein we found excellent agreement in the direction of change between isobaric labelling and label free quantification for at least 32 proteins which is approximately 90% of the common

proteins between the TMT changing list and the label free identified list (Supplementary Fig. S6).

RNA-seq

RNA sequencing

Using Illumina's TruSeq RNA Sample Prep v2 kits, poly-A tailed RNA (mRNA) was purified from total RNA using an oligo dT magnetic bead pull-down. The mRNA was then fragmented using metal ion-catalyzed hydrolysis. A random-primed cDNA library was then synthesised and this resulting double-strand cDNA was used as the input to a standard Illumina library prep: ends were repaired with a combination of fill-in reactions and exonuclease activitiy to produce blunt ends. A-tailing was performed, whereby an "A" base was added to the blunt ends followed by ligation to Illumina Paired-end Sequencing adapters containing unique index sequences, allowing samples to be pooled. The libraries then went through 10 cycles of PCR amplification using KAPA Hifi Polymerase rather than the kit-supplied Illumina PCR Polymerase due to better performance.

Samples were quantified and pooled based on a post-PCR Agilent Bioanalyzer, then the pool was size-selected using the LabChip XT Caliper. The multiplexed library was then sequenced on the Illumina HiSeq 2000, 75bp paired-end read length. Sequenced data was then analysed and quality controlled, and individual indexed library BAM files were produced.

Read alignment

The resulting reads that passed QC were realigned to the GRCh37 assembly of the human genome using a splice-aware aligner, tophat2 [31], and using a reference transcriptome from Ensembl release 75 [32], using the –library-type option fr-firststrand to bowtie. We limited the alignments to uniquely mapping reads. We then counted the number of reads aligning to each gene in the reference transcriptome using htseq-count from the HTSeq package [33] separately for each sample to produce a read count matrix counting the number of reads mapping to each gene in the transcriptome for each sample. To quantify absolute transcript abundance we computed the fragments per kilobase of transcript per million fragments mapped (FPKM) [10] for each gene using the total read counts from this matrix, and the exonic length of each gene calculated from gene models from Ensembl release 75. We obtained a mean of 49.3 million uniquely mapping reads from each sample (range: 39.2-71.4 million) with a mean of 84% of reads mapping to genes (range: 67.9-90.6%) which were used for the differential expression analysis.

Differential expression analysis

We used edgeR version 3.0 [34] to identify differentially expressed genes from the read count matrix. We restricted the analysis to 15,418 genes with >1 counts per million in at least 3 samples (similar to the protocol described by [35]). We followed the processing steps listed in the manual, using a generalized linear model with tissue status (degraded or intact) and individual ID as covariates. 349 genes were differentially expressed between the degraded and intact samples at 5% FDR (296 up-, 54 down-regulated in degraded tissue). The genes differentially expressed at 5% FDR had somewhat higher exonic length than the remaining genes (Wilcox-test p=0.00013; 4804 vs 4153 bases), hence we adjusted for gene length in the randomizations for gene set analyses (see below).

Methylation

Illumina 450k BeadChip assay

Sample submission

Samples were tested for quality and then quantified to 50ng/ul by the onsite sample management team prior to submission to the Illumina Genotyping pipeline. Before

processing begins, manifests for submitted samples are uploaded to Illumina LIMS where each sample plate is assigned an identification batch so that it can be tracked throughout the whole process that follows.

Bisulfite Conversion
Before Pre-Amplification sample DNA requires bisulfite conversion using the Zymo EZ-96 DNA Methylation assay. This is completed manually as per Zymo SOP guidelines.

Pre-Amplification
Due to the differences in sample plates between the completed Zymo assay and the Illumina assay, pre-Amplification is performed manually following the Illumina MSA4 SOP. Once complete, sample and reagent barcodes are scanned through the Illumina LIMS tracking software. Four micro-litres (200ng) of sample is required (Illumina guidelines) for the pre-Amplification reaction – there is no quantification step after the completion of the Zymo assay.

Post-Amplification
Over three days, Post-Amplification (Fragmentation, Precipitation, Resupension, Hybrisation to beadchip and xStaining) processes are completed as per Illumina protocol using four Tecan Freedom Evos. Following the staining process, BeadChips are coated for protection and dried completely under vacuum before scanning commences on five Illumina iScans, four of which are paired with two Illumina Autloader 2.Xs.

Image Beadchip
The iScan Control software determines intensity values for each bead type on the BeadChip and creates data files for each channel (.idat). Genomestudio uses this data file in conjunction with the beadpool manifest (.bpm) to analysis the data from the assay.

Quality Control
Prior to downstream analysis, all samples undergo an initial QC to establish how successful the assay has performed. Intensity graphs in Genomestudio's Control Dashboard identify sample performance by measuring dependent and non-dependent controls that are manufactured onto each BeadChip during production.

Probe-level analysis
The intensity files for each sample were processed using the ChAMP package [36]. Probes mapping to chromosomes X & Y, and those with a detection p-value >0.01 (n=3,064) were excluded. The beta values for each probe were quantile-normalized, accounting for the design of the array, using the 'dasen' method from the wateRmelon package [37]. We also excluded any probes with a common SNP (minor allele frequency >5%) within 2 base pairs of the CpG site, and those predicted to map to multiple locations in the genome [38] (n=45,218), leaving a total of 425,694 probes for the probe-level differential methylation analysis. We annotated all probes with genomic position, gene and genic location information from the ChAMP package.

To identify probes with evidence of differential methylation we used the CpGassoc package [39] to fit a linear model at each probe, with tissue status and individual ID as covariates. This analysis yielded 9,867 differentially methylated probes (DMP) between degraded and intact samples at 5% FDR.

Region-level analysis

To identify differentially methylated regions, we used custom software (available upon request) to identify regions containing at least 3 DMPs and no more than 3 non-significant probes with no more than 1kb between each constituent probe, following previous analyses [40]. We used bedtools [41] to identify genes overlapping each DMR, using gene annotations from Ensembl release 75, and extending each gene's bound to include 1500 basepairs upstream of the transcription start site to include likely promoter regions. This analysis yielded 271 DMRs with a mean of 4.04 DMPs per region, and a mean length of 673 basepairs.

Promoter-level analysis

We assigned probes in the promoter region of each gene using the probe annotations from the ChAMP package, and assigned to each gene any probe with the annotation "TSS1500", "TSS200", "5'UTR" and "1stExon" in order to capture probes in likely promoter regions. We then computed the mean normalized beta value of assigned probes for all genes with at least 5 associated probes for each sample separately, to produce a single methylation value for each gene in each sample. We used a paired t-test to identify genes with differential promoter-region methylation between degraded and intact samples, and a 5% FDR cutoff to call a gene's promoter region as differentially methylated. Note that the paired t-test assumes an equivalent model to the linear model used for the probe-level analysis.

Immunohistochemistry

To identify whether native chondrocytes demonstrated expression of the key factors immunohistochemistry was deployed. Four micron sections were dewaxed, rehydrated, and endogenous peroxidase blocked using 3% hydrogen peroxide for 30 minutes. After washing sections with dH_2O, antigens were retrieved in 0.01% w/v chymotrypsin/$CaCl_2$ (Sigma, UK), for 30 minutes at 37˚C. Following TBS washing, nonspecific binding sites were blocked at room temperature for 2 hours with either 25% w/v goat serum or rabbit serum (Abcam, UK) in 1% w/v bovine serum albumin (Sigma, UK) in TBS. Sections were incubated overnight at 4˚C with either mouse monoclonal primary antibodies or rabbit polyclonal antibodies. Negative controls in which rabbit and mouse IgGs (Abcam, UK) replaced the primary antibody at an equal protein concentration were used. Slides were washed in TBS and a biotinylated secondary antibody was applied; either goat anti-rabbit or rabbit anti-mouse, both antibodies were applied at 1:400 dilution in 1% w/v BSA/TBS for 30 minutes at room temperature. Binding of the secondary antibody was disclosed with streptavidin-biotin complex (Vector Laboratories, UK) technique with 0.08% v/v hydrogen peroxide in 0.65 mg/mL 3,3'-diaminobenzidine tetrahydrochloride (Sigma, UK) in TBS. Sections were counterstained with Mayer's haematoxylin (Leica, UK), dehydrated, cleared and mounted with Pertex (Leica, UK). All slides were visualised using an Olympus BX60 microscope and images captured using a digital camera and software program QCapture Pro v8.0 (MediaCybernetics, UK).

Protein atlas annotation

We downloaded annotations from Human Protein Atlas version 13, and annotated each protein-coding gene from the 3 experiments with the following terms taken from the annotation file: "Predicted secreted protein", "Predicted membrane protein", "Plasma protein". The secreted and membrane protein predictions are based on a consensus call from multiple computational prediction algorithms, and the plasma protein annotations are taken from the Plasma Protein Database, as detailed in [9].

Identification of previously reported OA genes

In order to identify whether some of the genes we highlight have previously been reported as associated with OA, we searched PubMed on 2 September 2016. We used an "advanced" search of the form "(osteoarthritis) AND (<gene_name>)" where <gene_name> was set to each HGNC gene symbol and we report the number of citations returned for each search.

Replication of gene expression changes using RNA-seq data and replication of methylation changes

Knee samples

Tissue samples were collected under National Research Ethics approval reference 15/SC/0132, South Yorkshire and North Derbyshire Musculoskeletal Biobank, University of Sheffield. All samples were collected from patients undergoing total knee replacement for primary osteoarthritis, and all patients provided written informed consent before participation. The patients comprised 12 women and 5 men, mean age 71 years (range 54-82). Patients with diagnosis other than osteoarthritis were excluded from the study. All sample processing steps (extraction of chondrocytes, extraction of DNA) were carried out as for the knee OA discovery samples.

Hip samples

Tissue samples were collected under National Research Ethics approval reference 11/EE/0011, Cambridge Biomedical Research Centre Human Research Tissue Bank, Cambridge University Hospitals, UK. All samples were collected from patients undergoing total hip replacement for osteoarthritis. The patients, who provided written consent before participation, comprised 6 women and 3 men, mean age 61 years (range 44-84). Osteoarthritis was confirmed by examination of the excised femoral head. Cartilage tissue was classified macroscopically and visually: low-grade cartilage as cartilage with a smooth surface with no obvious evidence of damage or fibrillation, high-grade cartilage as damaged and fibrillated cartilage usually occurring around eburnated areas of exposed bone. All sample processing steps (extraction of chondrocytes, extraction of DNA) were carried out as for the knee OA discovery samples, except that the cartilage was digested overnight in 6mg/ml collagenase A (Roche) in medium containing 10% serum to release the cells.

RNA-seq data

We applied the same procedure to the knee and hip replication data as to the discovery data. We considered 14762 genes that passed QC in the knee discovery, knee replication, and hip replication data; this included 332 of 349 genes with FDR<=5% in the knee discovery data.

Methylation

We used the Illumina 450k BeadChip to assay methylation for all knee and hip replication samples, using the same procedure as for the knee discovery data. The knee and hip methylation data were processed using the same QC procedure as for the knee discovery samples, including the same QC thresholds. We excluded one degraded hip cartilage sample as an outlier in probe failure rate (over six times the proportion of the next highest sample), and also excluded the paired intact cartilage sample. After QC, 417077 probes remained. We considered 416437 probes with methylation values in the knee discovery, knee replication, and hip replication data; this included 9723 of 9867 probes with FDR<=5% in the knee discovery data ("DMPs").

Microarray data

OA-dependent changes in expression of genes with differential expression in the discovery data were assessed with the help of an available dataset from the ongoing Research Arthritis and Articular Cartilage (RAAK) study, consisting in gene expression profiles of OA affected cartilage and macroscopically preserved cartilage from 33 patients undergoing total joint replacement surgery (22 with hip OA, 11 with knee OA). Sample collection and determination of gene expression levels have been described in detail previously [11]. In short, cartilage was collected separately for the OA affected and the unaffected regions of the weight baring part of the joint, snap frozen in liquid nitrogen and stored at -80°C prior to RNA extraction. Gene expression was determined with the Illumina HumanHT-12 v3 microarrays. After removal of probes that were not optimally measured (detection p-value >0.05 in more than 50% of the samples) a paired t-test was performed on all sample pairs while adjusting for chip (to adjust for possible batch effects).

Gene set analyses

Individual datasets

We aimed to test whether particular biological gene sets were enriched among the significant genes from each of the RNA-seq, methylation, and proteomics datasets. We used KEGG [42] and Reactome [43] gene annotations from MSigDB (v4) [44]. We also downloaded Gene Ontology (GO) biological process and molecular function gene annotations from QuickGO [45] on 4 February 2015. For GO, we only considered annotations with evidence codes IMP, IPI, IDA, IEP, and TAS. Genes annotated to the same term were treated as a "pathway". KEGG/Reactome and GO annotations were analysed separately and only pathways with 20 to 200 genes were considered (555 for KEGG/Reactome, 677 for GO).

Enrichment was assessed using a 1-sided hypergeometric test and only considering genes with annotations from a particular resource. For example, among the 15418 genes with RNA sequencing data, 4787 genes had KEGG/Reactome annotations, and 65 genes were annotated to "extracellular matrix annotation" in KEGG. Of the 350 significantly differentially expressed genes, 134 had KEGG/Reactome annotations, and 12 genes were annotated to "extracellular matrix annotation". Consequently, the enrichment of "extracellular matrix annotation" genes among the differentially expressed genes was assessed by comparing 12 of 134 to 65 of 4787 genes. Multiple-testing was accounted for by using a 5% FDR (separately for KEGG/Reactome and GO, and for RNA-seq, methylation, and protein expression data).

Empirical p-values for the enrichments were obtained from randomisations accounting for overlap of significant genes among the RNA-seq, methylation, and protein expression datasets were carried out by subdividing genes into bins as described for integrative analyses below.

Integrative gene set analyses

We aimed to integrate the gene sets analyses for the RNA-seq, methylation, and protein expression datasets. For each gene set, we asked whether the association across the three datasets (calculated as geometric mean of the *p*-values) was higher than expected by chance. To this end, we obtained 1-sided empirical *p*-values from 100,000 sets of "random RNA-seq genes, random methylation genes, and random protein expression genes". The "random" sets were chosen to conservatively match the overlap observed among the significant genes as follows. We performed the randomisation separately for KEGG/Reactome and for GO, as we only considered genes with at least one annotation in the resource.

To jointly construct one set each of random RNA-seq genes, random methylation genes, and random protein expression genes, we picked:

1) random genes for the overlap of RNA-seq, methylation, and protein expression (KEGG/Reactome: 2; GO: 3);
2) additional random genes for the overlap of RNA-seq and methylation (KEGG/Reactome: 4; GO: 10);
3) additional random genes for the overlap of RNA-seq and protein expression (KEGG/Reactome: 13; GO: 21);
4) additional random genes for the overlap of methylation and protein expression (KEGG/Reactome: 2; GO: 5);
5) additional RNA-seq random genes (KEGG/Reactome: 115; GO: 182);
6) additional methylation random genes (KEGG/Reactome: 73; GO: 102);
7) additional protein expression random genes (KEGG/Reactome: 62; GO: 113);

Random genes were picked to account for gene length as follows. In step 1, we subdivided all genes present in the RNA-seq, methylation, and protein expression data into 100 bins by increasing exonic length. If the original significant genes in the overlap had *g* genes in a particular bin *b*, we picked *g* random genes from that same bin; this was done for all 100 bins. Steps 2 to 7 were done analogously.

We tested that 100 bins were enough: choosing 50 or 200 bins gave very similar results (Pearson correlation >0.99 for empirical *p*-values in all enrichment analyses). We also confirmed that 10,000 random gene sets were enough: repeating the analysis gave very similar results (Spearman correlation >0.99 for empirical *p*-values in all enrichment analyses). To confirm the lower empirical *p*-values, we carried out 100,000 randomizations.

arcOGEN gene-set association analysis

We asked whether the 18 gene sets with strong evidence for association from the functional genomics data (Figure 4, Supplementary Fig. S4) are also associated with OA based on GWAS. To this end, we used the arcOGEN GWAS, primarily the 3498 cases with knee OA and all 11009 controls. We used MAGMA [46] v1.02 to carry out the gene-set analyses.
We assigned a SNP to a gene if it was located within the gene boundaries (Genome Assembly GRCh37). A SNP was assigned to a gene set if it was assigned to one of the genes in the given set.

First, we asked whether the average SNP *p*-value in a gene set was lower than expected by chance. We used the gene set test in plink, filtering independent SNPs at r^2=0.2, and 10000 case-control phenotype permutations to obtain empirical one-sided *p*-values. Five of the 18 gene sets had empirical *p*-values significant at 5% FDR (Supplementary Table S11). All of these five gene sets were also significantly associated at 5% FDR when considering all 7410 knee or/and hip OA cases and 11009 controls from arcOGEN, with similar results when filtering independent SNPs at r^2=0.5.

Second, we asked whether the results were confounded by population structure. To test this, we repeated the analysis accounting for population structure by using logistic regression with the 10 first principal components obtained from EIGENSTRAT when considering all 7410 cases and 11009 controls together with HapMap release 23a founder individuals. The results were as above (Supplementary Table S11).

Third, we asked whether the five gene sets with association in the first step were also associated compared to other gene sets, in particular, accounting for gene set size. We considered the 250 gene sets from GO, KEGG, and Reactome with the highest numbers of

SNPs assigned. For each of the five highlighted gene sets, we chose 100 gene sets with the closest numbers of assigned SNPs. At least one in ten of the other gene sets had empirical *p*-values as low as the highlighted gene set (Supplementary Table S11).

arcOGEN hypothesis-free gene-set analysis
We also asked whether we would have identified the 18 gene sets if we had only considered the arcOGEN knee OA GWAS data in a hypothesis-free approach. We used two common methods – a gene-based overrepresentation test as analogue to the functional genomics work, and a direct set-based test as above.

First, we asked whether the gene sets highlighted from the functional genomics work are among the gene sets over-represented among the 25% genes with the lowest *p*-values. We calculated *p*-values for each gene using plink gene set analysis as above. No gene set enrichment was significant at 5% FDR when considering all GO gene sets, and, separately, all KEGG and Reactome gene sets. (When considering all arcOGEN cases and controls, one GO and five KEGG/Reactome gene sets were significant at 5% FDR; they do not overlap with any of the 18 gene sets highlighted from the functional genomics analyses.)

Second, we asked whether the gene sets highlighted from the functional genomics work would have been among the significant results when all gene sets are analysed for low average SNP *p*-values. Here, we used the plink gene set test and chi-squared SNP *p*-values as above. Of the GO gene sets, 26 were significant at 5% FDR, including “platelet activation” and “ECM disassembly”, two of the largest gene sets highlighted from the functional genomics work. Of the KEGG/Reactome gene sets, 52 were significant at 5% FDR, including “signalling by PGDF”. All of these three gene sets had *q*-values >0.03 and were thus not among the most significant gene sets identified.

SUPPLEMENTARY TABLES

Supplementary Table S1. Full proteomics results.
Catalogue of total proteins identified in all TMT6plex experiments. The table includes UniProt Protein name (Protein), UniProt entry name (UniProt ID), GeneName, Log2Ratio Damaged/Control for each OA patient, median and standard deviation of all Log2Ratios, number of measurements (N), calculation of absolute Median- standard deviation (SD) value showing the consistency between different individuals compared to the median, and an indicator variable identifying if this protein was called as differentially abundant (diff_abundant).

Supplementary Table S2. Full RNA-seq differential expression edgeR analysis results.
Gene: gene name; logFC: log fold-change; logCPM: log counts per million; LR: likelihood ratio; PValue: p-value for differential expression; FDR: minimal false-discovery rate at which EmpPCombOneSided is significant.

Supplementary Table S3. Differential CpG probe methylation results.
Probe: Illumina probe identifier; chr: chromosome; pos: position of probe on GRCh37; mean_low_grade: mean beta value of all intact (low-grade) samples at this probe; mean_high_grade: mean beta value of all degraded (high-grade) samples at this probe; mean_beta: mean beta value of all samples at this probe; dbeta: difference in mean beta values between high and low-grade samples; gene_1: ChAMP package annotated gene; feature_1: ChAMP package annotation of gene region associated with gene_1; cgi: ChAMP package CpG island annotation; f_stat: F statistic for differential methylation; pval: p value for differential methylation; FDR: minimal FDR at which pval is significant.

Supplementary Table S4. Differentially methylated regions identified.
DMR: unique identifier for the differentially methylated region; chr: chromosome; start: (0-based) start coordinate on GRCh37; end: (1-based) end coordinate on GRCh37; num_DMPs: number of differentially methylated probes (DMP) in the DMR at 5% FDR; num_probes: total number of probes in the DMR (both DMP and non-DMP); mean_delta_beta: the mean difference in beta values of all probes in the DMR in degraded compared to intact cartilage samples; Genes: Ensembl gene name of overlapping genes (including 1.5kb promoter region upstream of each gene).

Supplementary Table S5. Genes with association on at least two molecular levels.
Gene symbol: the Ensembl gene name; DMR: -1 if the gene is associated with a DMR that is hypo-methylated in the degraded cartilage samples, 0 if the gene is not associated with a DMR, 1 if the gene is associated with a DMR that is hyper-methylated in the degraded cartilage samples; RNA: -1 if the gene is called as differentially expressed and is expressed at lower levels in the degraded cartilage samples, 0 if the gene is not called as differentially expressed, 1 if the gene is called as differentially expressed and is expressed at higher levels in the degraded cartilage samples; Proteomics: -1 if the protein is called as differentially adundant and is found at lower levels in the degraded cartilage samples, 0 if the protein is not called as differentially abundant, 1 if the protein is called as differentially abundant and is found at higher levels in the degraded cartilage samples; PubMed citations: number of citations returned for a PubMed search of the form "(osteoarthritis) AND (<gene symbol>)".

Supplementary Table S6. Gene expression replication results for genes with evidence of differential regulation from at least 2 experiments.

LogFC: log of fold-change; LogCPM: log counts per million; FDR: false-discovery rate (all columns from edgeR output). Direction Concord: concordance of direction of change ("1" for yes, "0" for no); Direction Concord RNA-seq and Repl p<=0.05: concordance of direction of change and at least nominal significance in the replication data ("1" for yes, "0" for no).

Supplementary Table S7. Promoter region methylation analysis results.

Gene: Ensembl gene name; mean_low_grade: mean beta value of probes in the promoter region of the gene in intact (low-grade) samples; mean_high_grade: mean beta value of probes in the promoter region of the gene in degraded (high-grade) samples; mean_beta: mean beta value of probes in the promoter region of the gene across all samples; delta_beta: the difference in mean beta values between degraded and intact cartilage samples; pval: p value for differential methylation; FDR: minimal FDR at which pval is significant.

Supplementary Table S8. Gene expression replication results for genes with significant change at 5% FDR in the knee discovery data.

Only genes present in the discovery and both knee and hip RNA-seq replication data are included.

LogFC: log of fold-change; LogCPM: log counts per million; FDR: false-discovery rate (all columns from edgeR output). Direction Concord: concordance of direction of change ("1" for yes, "0" for no); Direction Concord RNA-seq and Repl p<=0.05: concordance of direction of change and at least nominal significance in the replication data ("1" for yes, "0" for no).

Supplementary Table S9. KEGG/Reactome pathway enrichment results.

HyperGeomP: 1-sided hypergeometric enrichment p-value; ListPathGenes: genes with significant association from RNA/proteomics/methylation data that are annotated to given gene set; ListGenes: total number of genes with RNA/proteomics/methylation data that are annotated to given gene set; TotGenes: total number of genes with RNA/proteomics/methylation data that have gene annotations; EmpP: 1-sided empirical p-value for HyperGeomP.

Supplementary Table S10. GO term enrichment results.

Code: Gene Ontology annotation code; GODescr: Gene Ontology annotation description; GOtype: Gene Ontology annotation type (biological process or molecular function); HyperGeomP: 1-sided hypergeometric enrichment p-value; ListPathGenes: genes with significant association from RNA/proteomics/methylation data that are annotated to given gene set; ListGenes: total number of genes with RNA/proteomics/methylation data that are annotated to given gene set; TotGenes: total number of genes with RNA/proteomics/methylation data that have gene annotations; EmpP: 1-sided empirical p-value for HyperGeomP; EmpPCombOneSided: 1-sided empirical p-value for the geometric mean of the three hypergeometric p-values for the RNA, proteomics and methylation data; EmpPFDR: minimal false-discovery rate at which EmpPCombOneSided is significant.

Supplementary Table S11. Summary results from the arcOGEN GWAS pathway analysis. The 5 gene sets overlapping with those highlighted from the functional genomics experiments are highlighted in bold. Statistics are reported for analyses excluding and including principal components to account for population stratification (Methods). SET: gene set from GO or Kegg/Reactome; NSNP: number of SNPs in the gene set; ISNP: number of independent SNPs at r2<=0.2; EMP1: empirical p-value for gene set association (low average SNP p-value in set); FDR: minimal false-discovery rate at which EMP1 is significant. We also list a 'competitive' p-value for each of the 5 overlapping gene set p-values (knee OA, no PCA, 50000 phenotype permutations) to empirical p-values of 100 other gene sets of comparable size.

Supplementary Table S12. Down-regulated proteoglycans, cartilage and chondrocyte function-related proteins, and basement membrane proteins.
List of proteins (shown with gene names) consistently at lower abundances in OA degraded cartilage intact cartilage samples but not changing significantly in the RNA-seq data (with the exception of MATN4). The table shows Log2Ratio degraded/intact for each OA patient at protein and RNA levels as well as Uniprot function. The stacked column bars show the contribution of each value to the total protein and RNA levels of this group across the different individuals.

Supplementary Table S13. Ratios of chemiluminescence signal density between samples from each pair.
All density measurements were normalised to GAPDH loading control. n.d. indicates not detected; n.a. indicates not analysed.

Disease/Control (Density Normalised to loading control)				
	ANPEP	AQP1	TGFBI	WNT5B
SMPB049	8.43	3.09	1.22	2.51
SMPB051	3.43	2.66	1.96	2.32
SMPB054	3.55	5.21	2.73	4.24
SMPB055	0.36	n.d.	1.31	1.09
SMPB056	2.46	1.72	2.3	1.05
SMPB059	19.46	1.88	1.94	1.28
SMPB064	2.42	1.34	0.55	0.87
SMPB065	n.d.	n.a.	6.48	n.a.
	Increased	Increased	Increased	Increased

Supplementary Table S14. Results from genes proximal to GWAS signals.
SNP: genome-wide associated SNP; Gene: gene located within +/-500kb of the SNP and with at least one of RNA, proteomics, or methylation data; Dist: distance between SNP and gene (in bp); Methylation_logFC: mean log fold-change of gene promoter methylation; Methylation_MeanC: mean promoter methylation in control tissue; Methylation_p: p-value for differential gene promoter methylation; Methylation_FDR: minimal false-discovery rate at which Methylation_p is significant; RNA_logFC: log fold-change of gene RNA; RNA_logCPM: log counts per million of gene RNA; RNA_p: p-value for differential gene expression based on RNA; RNA_FDR: minimal false-discovery rate at which RNA_p is significant; Proteomics_median: median log of pairwise protein expression fold-change; Proteomics_N: number of samples in which protein was detected; Proteomics_|median|-SD: difference between absolute Median and standard deviation (SD) value showing the consistency between different individuals compared to the median (lower values mean more variation in protein change between sample pairs).

SUPPLEMENTARY FIGURES

Supplementary Figure S1. **Masson Trichrome stain of degraded (high-grade) and intact (low-grade) cartilage-bone interface.**

Cartilage is shown in blue, bone in red.

A) The stain demonstrates a healthy abundance of cartilage in the intact sample. A clear tidemark (----) that is not crossed by blood vessels can be seen.

B) The degraded cartilage sample shows clear depletion of cartilage and tissue remodeling. The tidemark (----) is disrupted, with blood vessel ingrowth that crossed the tidemark (arrows).

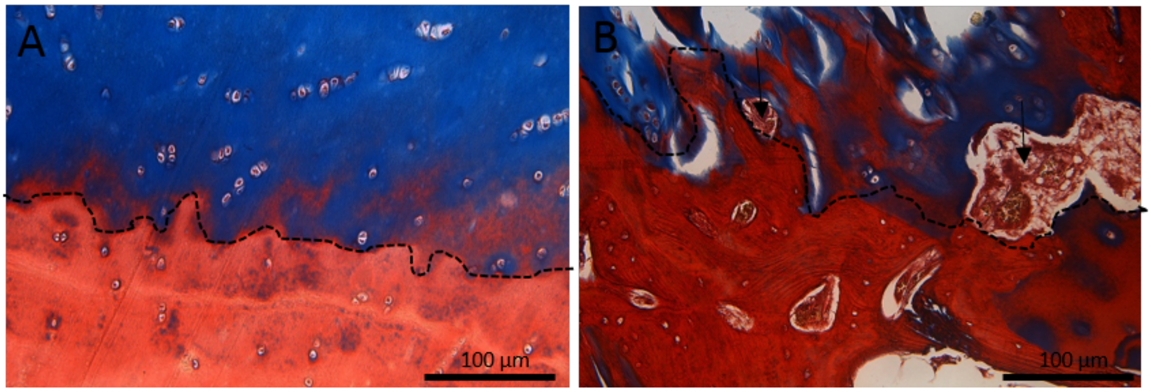

Supplementary Figure S2. Immunohistochemical identification of key proteins.

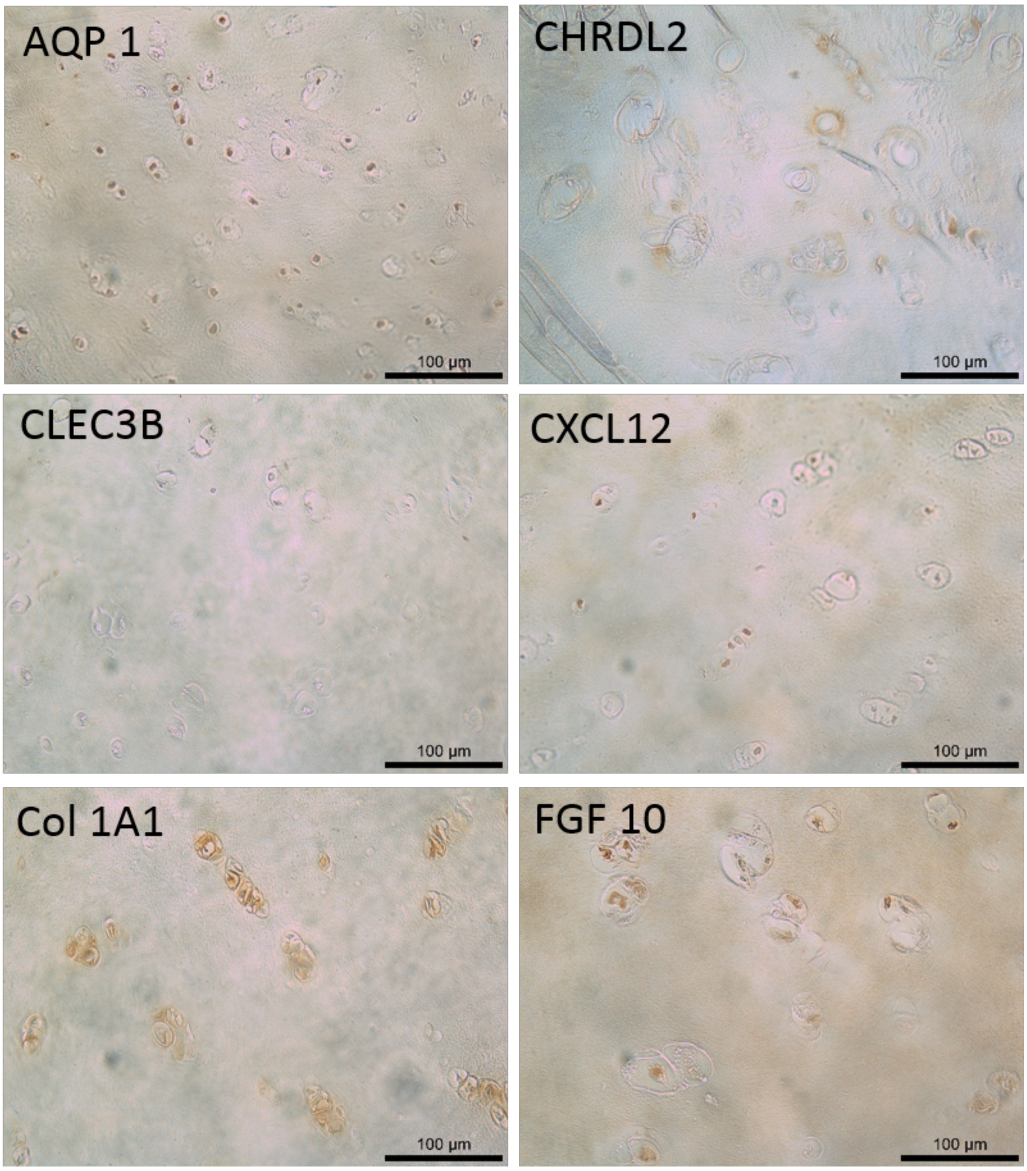

Supplementary Figure S3. Comparison of gene expression with protein abundance and with promoter region methylation.

a) A global comparison of gene expression (FPKM) and protein abundance (mean normalised spectral counts) for 4,095 protein-coding genes across all samples. The trend line is derived from a linear regression.

b) A global comparison of gene expression and promoter region methylation for 15,921 genes. The trend line is derived from a linear regression.

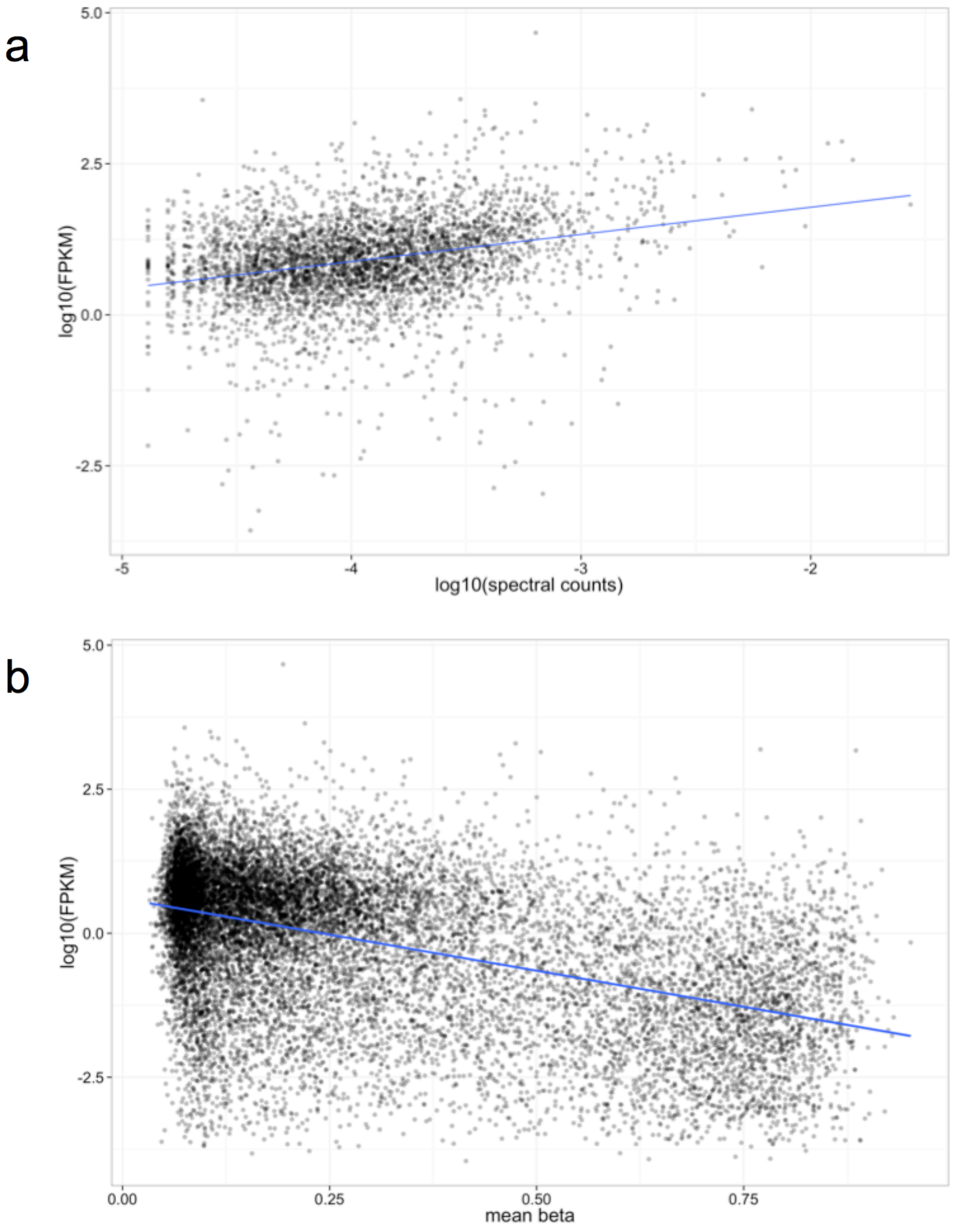

Supplementary Figure S4. **Significantly enriched gene sets in the integrative analysis.** a, b) Enrichments from KEGG/Reactome (a) and Gene Ontology (b). The circos plots show enriched gene sets, with genes differentially regulated in at least one of the methylation, RNA-seq, or proteomics experiments. Lines connect genes that occur in several gene sets. The three outside circles show boxes for genes with significantly higher (black) or lower (red) methylation, gene, or protein expression data. A red box with black border indicated a gene that overlaps hyper- as well as hypo-methylated DMRs.

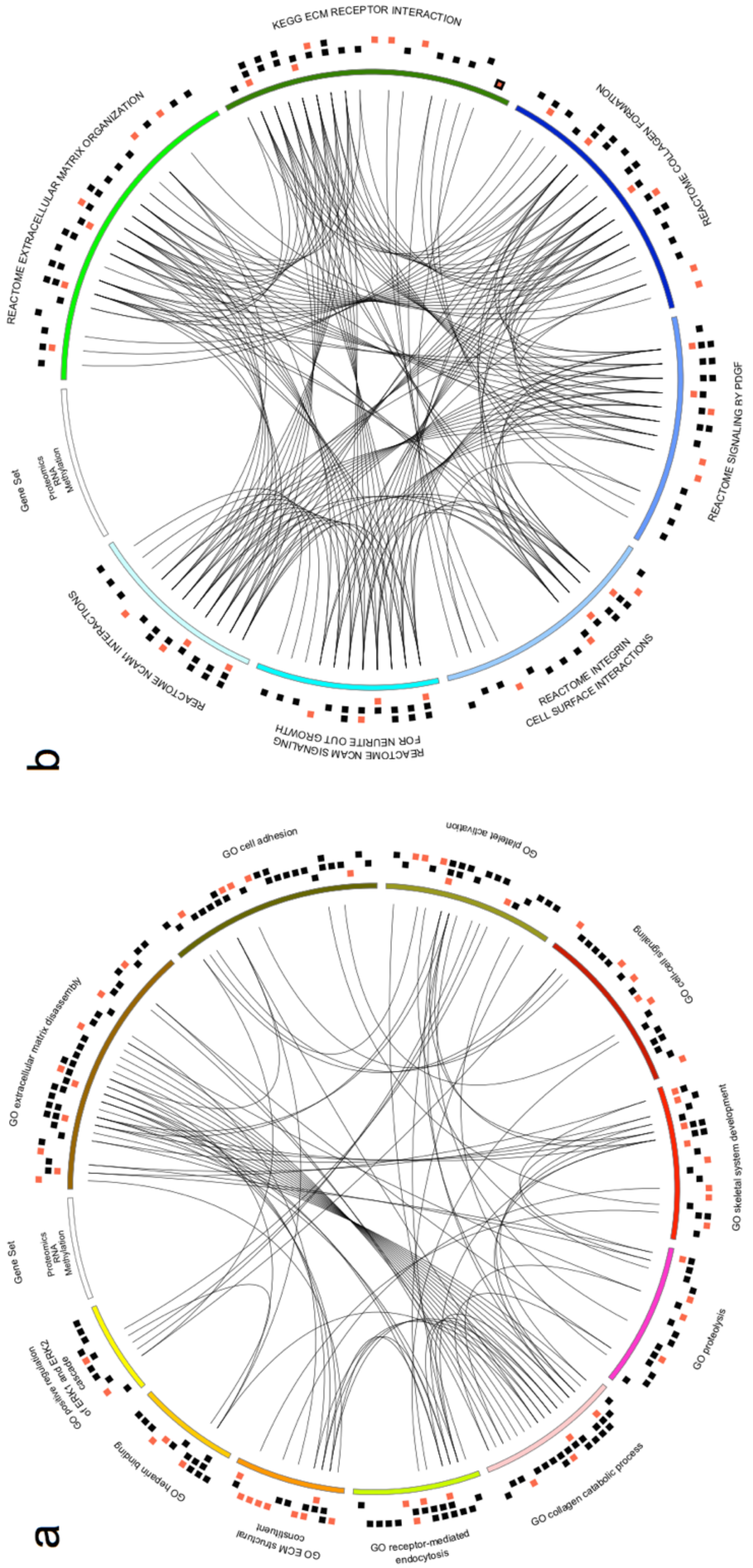

b
KEGG ECM RECEPTOR INTERACTION
REACTOME COLLAGEN FORMATION
REACTOME SIGNALING BY PDGF
REACTOME INTEGRIN CELL SURFACE INTERACTIONS
REACTOME NCAM SIGNALING FOR NEURITE OUT GROWTH
REACTOME NCAM1 INTERACTIONS
Gene Set
Proteomics
RNA
Methylation
REACTOME EXTRACELLULAR MATRIX ORGANIZATION
a
GO cell adhesion
GO platelet activation
GO cell-cell signaling
GO skeletal system development
GO proteolysis
GO collagen catabolic process
GO receptor-mediated endocytosis
GO ECM structural constituent
GO heparin binding
GO positive regulation of ERK1 and ERK2 cascade
Gene Set
Proteomics
RNA
Methylation
GO extracellular matrix disassembly

Supplementary Figure S5. **Expected discovery (EDR), true positive (TP) and true negative (TN) rates for calling differential gene expression (RNA) in the current dataset of 12 paired samples.**

The EDR estimates the proportion of truly differentially expressed genes that are identified as significant at the given p-value threshold. TP, TN and EDR were estimated using PowerAtlas [47]. The p-value thresholds represent: nominal significance (0.05); the threshold approximately corresponding to 5% FDR in this analysis (0.001); and genome-wide significance assuming 20,000 genes (2.5E-6).

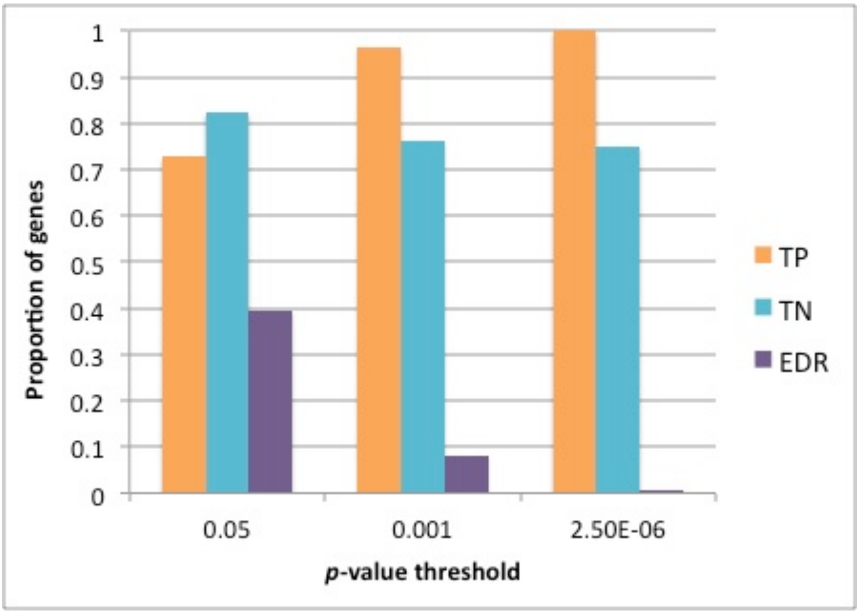

Supplementary Figure S6. Label free quantification of representative samples.
For scale normalization amongst the different proteins the spectral counts for each protein per sample were divided with the maximum value per protein. Bar charts were plotted based on the normalized spectral counts separately for the down-regulated (blue bars) and the up-regulated (red bars) proteins as found by TMT quantification. C stands for intact (low-grade cartilage) and D stands for degraded (high-grade) cartilage.

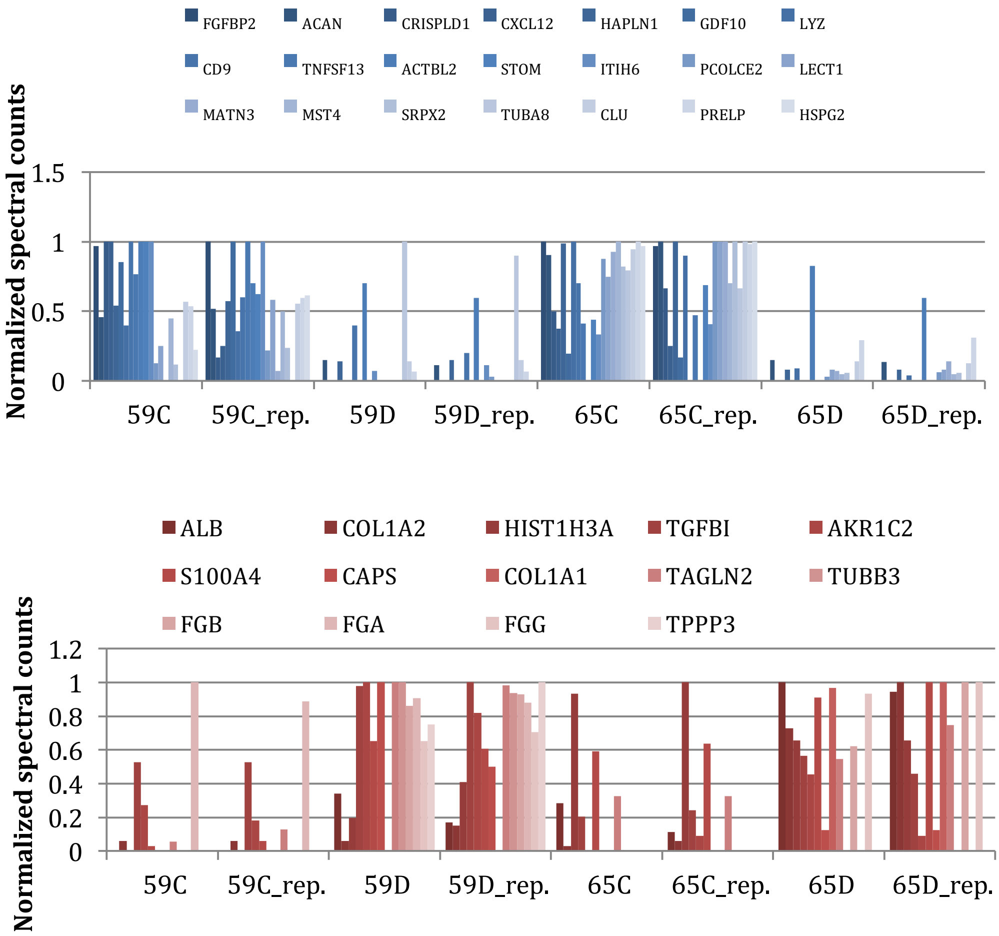

Supplementary Figure S7. Western blot of samples from eight individuals that were also analysed by mass spectrometry.

The number indicates the individual, C corresponds to intact (low-grade) and D to degraded (high-grade) sample pairs. Ratios of chemiluminescence signal density between samples from each pair are summarised in Supplementary Table S13.

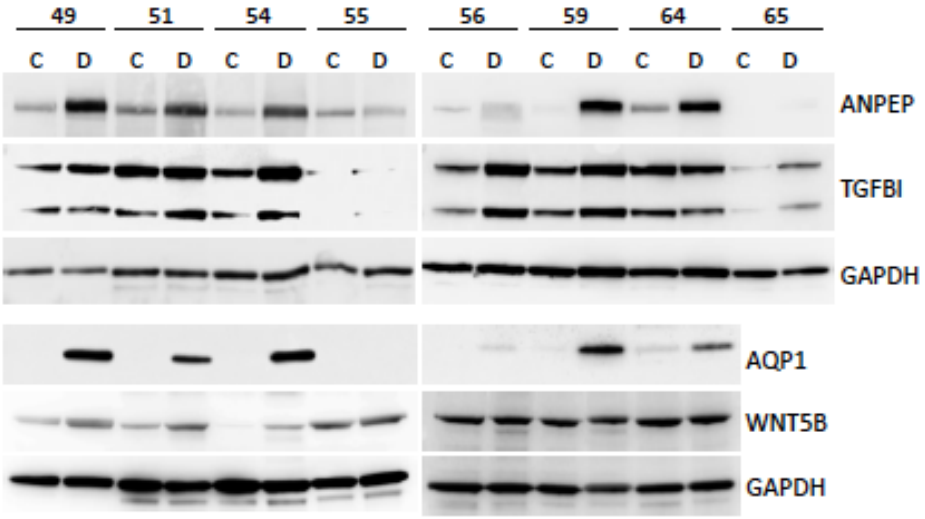

Supplementary Figure S8. Heatmap of the log fold change (in degraded compared to intact cartilage) showing the comparison between the TMT and Western blot based protein quantification for 8 individuals.

Red colour shows up-regulation and blue colour shows down-regulation in the degraded cartilage sample.

	TMT								Western Blot							
**Protein**	**SMP049**	**SMP051**	**SMP054**	**SMP055**	**SMP056**	**SMP059**	**SMP064**	**SMP065**	**SMP049**	**SMP051**	**SMP054**	**SMP055**	**SMP056**	**SMP059**	**SMP064**	**SMP065**
**ANPEP**	2.03	1.59	1.97	0.02	0.64	2.44	1.14	1.90	3.08	1.78	1.83	-1.47	1.30	4.28	1.28	n.a
**AQP1**	1.84	1.97	n.a	n.a	2.44	n.a	n.a	n.a	1.63	1.41	2.38	0.00	0.78	0.91	0.42	n.a
**WNT5B**	-0.28	-0.90	-0.78	-0.16	-0.83	-0.83	0.13	-0.73	1.33	1.21	2.08	0.12	0.07	0.36	-0.20	n.a
**TGFBI**	-0.46	0.15	1.37	0.80	1.07	0.94	0.06	1.14	0.50	1.16	1.54	1.84	1.20	0.96	-0.86	2.70

SUPPLEMENTARY REFERENCES

1. Lourido, L. *et al.* Quantitative Proteomic Profiling of Human Articular Cartilage Degradation in Osteoarthritis. *J. Proteome Res.* **13**, 6096-6106, doi:10.1021/pr501024p (2014).
2. Petrella, R. J. Hyaluronic Acid for the Treatment of Knee Osteoarthritis. *American Journal of Physical Medicine & Rehabilitation* **84**, 278-283, doi:10.1097/01.phm.0000156899.18885.06 (2005).
3. UniProt Consortium. Activities at the Universal Protein Resource (UniProt). *Nucleic Acids Research* **42**, 7486-7486, doi:10.1093/nar/gku469 (2014).
4. Mateos, J. *et al.* Differential protein profiling of synovial fluid from rheumatoid arthritis and osteoarthritis patients using LC–MALDI TOF/TOF. *Journal of Proteomics* **75**, 2869-2878, doi:10.1016/j.jprot.2011.12.042 (2012).
5. Attur, M. *et al.* Elevated expression of periostin in human osteoarthritic cartilage and its potential role in matrix degradation via matrix metalloproteinase-13. *The FASEB Journal* **29**, 4107-4121, doi:10.1096/fj.15-272427 (2015).
6. Kim, B. Y. *et al.* Corneal Dystrophy-associated R124H Mutation Disrupts TGFBI Interaction with Periostin and Causes Mislocalization to the Lysosome. *Journal of Biological Chemistry* **284**, 19580-19591, doi:10.1074/jbc.m109.013607 (2009).
7. Verma, P. & Dalal, K. ADAMTS-4 and ADAMTS-5: Key enzymes in osteoarthritis. *Journal of Cellular Biochemistry* **112**, 3507-3514, doi:10.1002/jcb.23298 (2011).
8. Song, R.-H. *et al.* Aggrecan degradation in human articular cartilage explants is mediated by both ADAMTS-4 and ADAMTS-5. *Arthritis Rheum* **56**, 575-585, doi:10.1002/art.22334 (2007).
9. Uhlen, M. *et al.* Tissue-based map of the human proteome. *Science* **347**, 1260419-1260419, doi:10.1126/science.1260419 (2015).
10. Trapnell, C. *et al.* Transcript assembly and quantification by RNA-Seq reveals unannotated transcripts and isoform switching during cell differentiation. *Nat Biotechnol* **28**, 511-515, doi:10.1038/nbt.1621 (2010).
11. Ramos, Y. F. M. *et al.* Genes Involved in the Osteoarthritis Process Identified through Genome Wide Expression Analysis in Articular Cartilage; the RAAK Study. *PLoS ONE* **9**, e103056, doi:10.1371/journal.pone.0103056 (2014).
12. Lee, A. S. *et al.* A current review of molecular mechanisms regarding osteoarthritis and pain. *Gene* **527**, 440-447, doi:10.1016/j.gene.2013.05.069 (2013).
13. Liu, Z., Cai, H., Zheng, X., Zhang, B. & Xia, C. The Involvement of Mutual Inhibition of ERK and mTOR in PLCγ1-Mediated MMP-13 Expression in Human Osteoarthritis Chondrocytes. *IJMS* **16**, 17857-17869, doi:10.3390/ijms160817857 (2015).
14. Otero, M. *et al.* E74-like Factor 3 (ELF3) Impacts on Matrix Metalloproteinase 13 (MMP13) Transcriptional Control in Articular Chondrocytes under Proinflammatory Stress. *Journal of Biological Chemistry* **287**, 3559-3572, doi:10.1074/jbc.m111.265744 (2012).
15. Prasadam, I., Zhou, Y., Shi, W., Crawford, R. & Xiao, Y. Role of dentin matrix protein 1 in cartilage redifferentiation and osteoarthritis. *Rheumatology* **53**, 2280-2287, doi:10.1093/rheumatology/keu262 (2014).
16. Xu, J., Yi, Y., Li, L., Zhang, W. & Wang, J. Osteopontin induces vascular endothelial growth factor expression in articular cartilage through PI3K/AKT and ERK1/2 signaling. *Mol Med Rep* **12**, 4708-4712, doi:10.3892/mmr.2015.3975 (2015).
17. Chia, S.-L. *et al.* Fibroblast growth factor 2 is an intrinsic chondroprotective agent that suppresses ADAMTS-5 and delays cartilage degradation in murine osteoarthritis. *Arthritis Rheum* **60**, 2019-2027, doi:10.1002/art.24654 (2009).

18 Long, D. L., Ulici, V., Chubinskaya, S. & Loeser, R. F. Heparin-binding epidermal growth factor-like growth factor (HB-EGF) is increased in osteoarthritis and regulates chondrocyte catabolic and anabolic activities. *Osteoarthritis and Cartilage* **23**, 1523-1531, doi:10.1016/j.joca.2015.04.019 (2015).

19 Patil, A. S., Sable, R. B. & Kothari, R. M. Occurrence, biochemical profile of vascular endothelial growth factor (VEGF) isoforms and their functions in endochondral ossification. *J. Cell. Physiol.* **227**, 1298-1308, doi:10.1002/jcp.22846 (2012).

20 Pufe, T., Groth, G., Goldring, M. B., Tillmann, B. & Mentlein, R. Effects of pleiotrophin, a heparin-binding growth factor, on human primary and immortalized chondrocytes. *Osteoarthritis and Cartilage* **15**, 155-162, doi:10.1016/j.joca.2006.07.005 (2007).

21 Marmotti, A. *et al.* PRP and Articular Cartilage: A Clinical Update. *BioMed Research International* **2015**, 1-19, doi:10.1155/2015/542502 (2015).

22 Meheux, C. J., McCulloch, P. C., Lintner, D. M., Varner, K. E. & Harris, J. D. Efficacy of Intra-articular Platelet-Rich Plasma Injections in Knee Osteoarthritis: A Systematic Review. *Arthroscopy: The Journal of Arthroscopic & Related Surgery* **32**, 495-505, doi:10.1016/j.arthro.2015.08.005 (2016).

23 Zhou, Q. *et al.* Platelets promote cartilage repair and chondrocyte proliferation via ADP in a rodent model of osteoarthritis. *Platelets* **27**, 212-222, doi:10.3109/09537104.2015.1075493 (2015).

24 Ashraf, S. & Walsh, D. A. Angiogenesis in osteoarthritis. *Current Opinion in Rheumatology* **20**, 573-580, doi:10.1097/bor.0b013e3283103d12 (2008).

25 Mapp, P. I. & Walsh, D. A. Mechanisms and targets of angiogenesis and nerve growth in osteoarthritis. *Nat Rev Rheumatol* **8**, 390-398, doi:10.1038/nrrheum.2012.80 (2012).

26 Evangelou, E. *et al.* A meta-analysis of genome-wide association studies identifies novel variants associated with osteoarthritis of the hip. *Annals of the Rheumatic Diseases* **73**, 2130-2136, doi:10.1136/annrheumdis-2012-203114 (2013).

27 Panoutsopoulou, K. & Zeggini, E. Advances in osteoarthritis genetics. *Journal of Medical Genetics* **50**, 715-724, doi:10.1136/jmedgenet-2013-101754 (2013).

28 arcOGEN Consortium. Identification of new susceptibility loci for osteoarthritis (arcOGEN): a genome-wide association study. *The Lancet* **380**, 815-823, doi:http://dx.doi.org/10.1016/S0140-6736(12)60681-3 (2012).

29 Mankin, H. J., Dorfman, H., Lippiello, L. & Zarins, A. Biochemical and metabolic abnormalities in articular cartilage from osteo-arthritic human hips. II. Correlation of morphology with biochemical and metabolic data. *The Journal of bone and joint surgery. American volume* **53**, 523-537 (1971).

30 Pearson, R. G., Kurien, T., Shu, K. S. S. & Scammell, B. E. Histopathology grading systems for characterisation of human knee osteoarthritis – reproducibility, variability, reliability, correlation, and validity. *Osteoarthritis and Cartilage* **19**, 324-331, doi:10.1016/j.joca.2010.12.005 (2011).

31 Kim, D. *et al.* TopHat2: accurate alignment of transcriptomes in the presence of insertions, deletions and gene fusions. *Genome Biology* **14**, R36, doi:10.1186/gb-2013-14-4-r36 (2013).

32 Flicek, P. *et al.* Ensembl 2014. *Nucleic Acids Research* **42**, D749-D755, doi:10.1093/nar/gkt1196 (2013).

33 Anders, S., Pyl, P. T. & Huber, W. HTSeq--a Python framework to work with high-throughput sequencing data. *Bioinformatics* **31**, 166-169, doi:10.1093/bioinformatics/btu638 (2014).

34 Robinson, M. D., McCarthy, D. J. & Smyth, G. K. edgeR: a Bioconductor package for differential expression analysis of digital gene expression data. *Bioinformatics* **26**, 139-140, doi:10.1093/bioinformatics/btp616 (2009).
35 Anders, S. *et al.* Count-based differential expression analysis of RNA sequencing data using R and Bioconductor. *Nat Protoc* **8**, 1765-1786, doi:10.1038/nprot.2013.099 (2013).
36 Morris, T. J. *et al.* ChAMP: 450k Chip Analysis Methylation Pipeline. *Bioinformatics* **30**, 428-430, doi:10.1093/bioinformatics/btt684 (2014).
37 Pidsley, R. *et al.* A data-driven approach to preprocessing Illumina 450K methylation array data. *BMC Genomics* **14**, 293, doi:10.1186/1471-2164-14-293 (2013).
38 Chen, Y.-a. *et al.* Discovery of cross-reactive probes and polymorphic CpGs in the Illumina Infinium HumanMethylation450 microarray. *Epigenetics* **8**, 203-209, doi:10.4161/epi.23470 (2013).
39 Barfield, R. T., Kilaru, V., Smith, A. K. & Conneely, K. N. CpGassoc: an R function for analysis of DNA methylation microarray data. *Bioinformatics* **28**, 1280-1281, doi:10.1093/bioinformatics/bts124 (2012).
40 den Hollander, W. *et al.* Knee and hip articular cartilage have distinct epigenomic landscapes: implications for future cartilage regeneration approaches. *Annals of the Rheumatic Diseases* **73**, 2208-2212, doi:10.1136/annrheumdis-2014-205980 (2014).
41 Quinlan, A. R. & Hall, I. M. BEDTools: a flexible suite of utilities for comparing genomic features. *Bioinformatics* **26**, 841-842, doi:10.1093/bioinformatics/btq033 (2010).
42 Kanehisa, M. KEGG: Kyoto Encyclopedia of Genes and Genomes. *Nucleic Acids Research* **28**, 27-30, doi:10.1093/nar/28.1.27 (2000).
43 Matthews, L. *et al.* Reactome knowledgebase of human biological pathways and processes. *Nucleic Acids Research* **37**, D619-D622, doi:10.1093/nar/gkn863 (2009).
44 Subramanian, A. *et al.* Gene set enrichment analysis: A knowledge-based approach for interpreting genome-wide expression profiles. *Proceedings of the National Academy of Sciences* **102**, 15545-15550, doi:10.1073/pnas.0506580102 (2005).
45 Binns, D. *et al.* QuickGO: a web-based tool for Gene Ontology searching. *Bioinformatics* **25**, 3045-3046, doi:10.1093/bioinformatics/btp536 (2009).
46 de Leeuw, C. A., Mooij, J. M., Heskes, T. & Posthuma, D. MAGMA: Generalized Gene-Set Analysis of GWAS Data. *PLoS Comput Biol* **11**, e1004219, doi:10.1371/journal.pcbi.1004219 (2015).
47 Page, G. P. *et al.* The PowerAtlas: a power and sample size atlas for microarray experimental design and research. *BMC Bioinformatics* **7**, 84, doi:10.1186/1471-2105-7-84 (2006).
